# Varicella Zoster Virus Infection: Clinical Features, Molecular Pathogenesis, Treatment, and Prevention

**DOI:** 10.1002/mco2.70661

**Published:** 2026-03-05

**Authors:** Lei Peng, Honghao Song, Tianying Li, Yuqing Ma, Chen Yan, Yuhan Cao, Kaiqiang Sun, Chaofeng Han, Hongbin Yuan

**Affiliations:** ^1^ Department of Anesthesiology Changzheng Hospital, Naval Medical University Shanghai China; ^2^ School of Basic Medical Science Naval Medical University Shanghai China; ^3^ Nautical Medicine Experimental Teaching Demonstration Center of Educational Institutions Faculty of Naval Medicine Navy Medical University Shanghai China; ^4^ Department of Orthopedic Surgery Changzheng Hospital, Navy Medical University Shanghai China; ^5^ Department of Orthopedics Naval Medical Center of PLA Shanghai China; ^6^ Department of Histology and Embryology Naval Medical University Shanghai China; ^7^ National Key Laboratory of Immunity & Inflammation Naval Medical University Shanghai China

**Keywords:** varicella zoster virus, aging, clinical management, pathogenesis, reactivation, prevention, research translation

## Abstract

Varicella zoster virus (VZV) is a ubiquitous human herpesvirus that establishes lifelong latency and causes herpes zoster (HZ) upon reactivation, posing a growing clinical challenge in aging populations. The incidence of HZ increases sharply with age and immunocompromised states, and its clinical burden extends well beyond cutaneous disease to include postherpetic neuralgia, neurological complications, and systemic involvement. At the mechanistic level, HZ reflects a dynamic interplay between viral reactivation, host immune surveillance, and virus‐induced neuronal injury. Despite substantial progress in antiviral therapy and vaccination, many interventions remain focused on individual stages of the disease process, and the translation of molecular insights into comprehensive clinical strategies remains incomplete. In this review, we synthesize current knowledge of VZV epidemiology, structure, and life cycle, and provide an integrated overview of clinical manifestations and molecular pathogenesis, with particular emphasis on latency, reactivation, and immune evasion. We further summarize advances in diagnostic technologies and discuss therapeutic strategies targeting viral replication, inflammatory complications, and long‐term sequelae, alongside preventive approaches. By linking disease mechanisms with clinical management, this review highlights key challenges and emerging directions for improving the prevention and treatment of HZ and provides a framework for translating fundamental discoveries into more effective interventions.

## Graphical abstract

This review focuses on the clinical features, molecular mechanisms, treatment methods, and preventive measures of the VZV, with the aim of providing a theoretical basis for the development of new treatment and prevention methods for HZ.

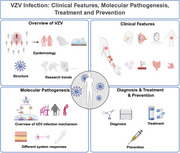

## 1 Introduction

For many decades, varicella (chickenpox) and herpes zoster (HZ) were regarded as clinically distinct entities [1]. This view was fundamentally revised in 1953, when Thomas Weller successfully isolated the causative virus responsible for both conditions, thereby establishing varicella zoster virus (VZV) [2] as their shared etiological agent. This discovery enabled the formulation of the classical model of VZV pathogenesis, in which primary infection leads to lifelong latency in sensory ganglia, followed by viral reactivation later in life [2]. Globally, HZ remains a substantial public health burden, with an incidence of approximately three to five cases per 1000 person‐years. Hospitalization and mortality rates range from 2 to 25 and 0.017 to 0.465 per 100,000 person‐years, respectively, and recurrence occurs in 1–10% of cases [3]. Unlike acute respiratory viral infections, VZV establishes lifelong persistence within the host nervous system [4]. Reactivation, driven by advanced age [3, 5], immunosenescence [6, 7], or physiological stress [8], results in HZ, which is typically characterized by a painful, dermatomal rash [2].

As a member of the Alphaherpesvirinae subfamily, VZV is an enveloped virus with a double‐stranded Deoxyribonucleic acid (DNA) Human immunodeficiency virus genome of approximately 125 kilobases [9, 10]. Although only one serotype exists, the virus can be classified into genotypes based on polymorphisms in genes such as ORF22 and ORF62 [10, 11]. These genotypes exhibit minimal differences in virulence and do not affect cross‐protection [11]. As an exclusively human pathogen, VZV spreads through the inhalation of aerosolized droplets or direct contact with fluid from vesicles [12]. The consequence of primary VZV infection is varicella, with subsequent retrograde transport of the virus to sensory ganglia for the establishment of latency [12]. Reactivation, frequently associated with declining cell‐mediated immunity, results in viral transit along sensory nerves to the dermatome, producing the characteristic unilateral rash and neuropathic pain of HZ [12]. The risk is markedly elevated in older adults and immunocompromised individuals [3, 7].

The approval of acyclovir (ACV) in 1981 marked a therapeutic milestone as the first effective oral antiherpesviral agent [13, 14]. However, its low oral bioavailability (10–20%) prompted the development of improved analogs such as famciclovir (FAM) [15] and valacyclovir (VACV) [16], which offer superior pharmacokinetics. Concurrently, growing emphasis has been placed on managing HZ complications, with multimodal analgesia forming the cornerstone of therapy for neuropathic pain [17]. In parallel, vaccination has emerged as the most cost‐effective public health strategy for VZV control [18]. The live‐attenuated zoster vaccine (ZVL), licensed in 2006 [19, 20], was later supplemented by the recombinant zoster vaccine (RZV) in 2017 [21]. Despite these advances, most available interventions target only a single stage of the VZV lifecycle, and a significant translational gap remains in converting pathophysiological insights into clinical applications that address the full disease spectrum.

To provide a comprehensive framework for understanding the treatment and prevention of HZ, this review first summarizes the structure, epidemiology, and evolving research landscape of VZV. We then integrate current knowledge on clinical manifestations, molecular pathogenesis, and diagnostic strategies, with particular emphasis on recent advances in antiviral therapy and vaccination. Preventive approaches are discussed across multiple platforms, including live‐attenuated, recombinant subunit, viral‐vectored, and nucleic acid‐based vaccines. Finally, we highlight key translational challenges and emerging directions in VZV research, aiming to bridge fundamental mechanisms with improved clinical management.

## 2 Overview of VZV

### 2.1 Epidemiology

**FIGURE 1:**
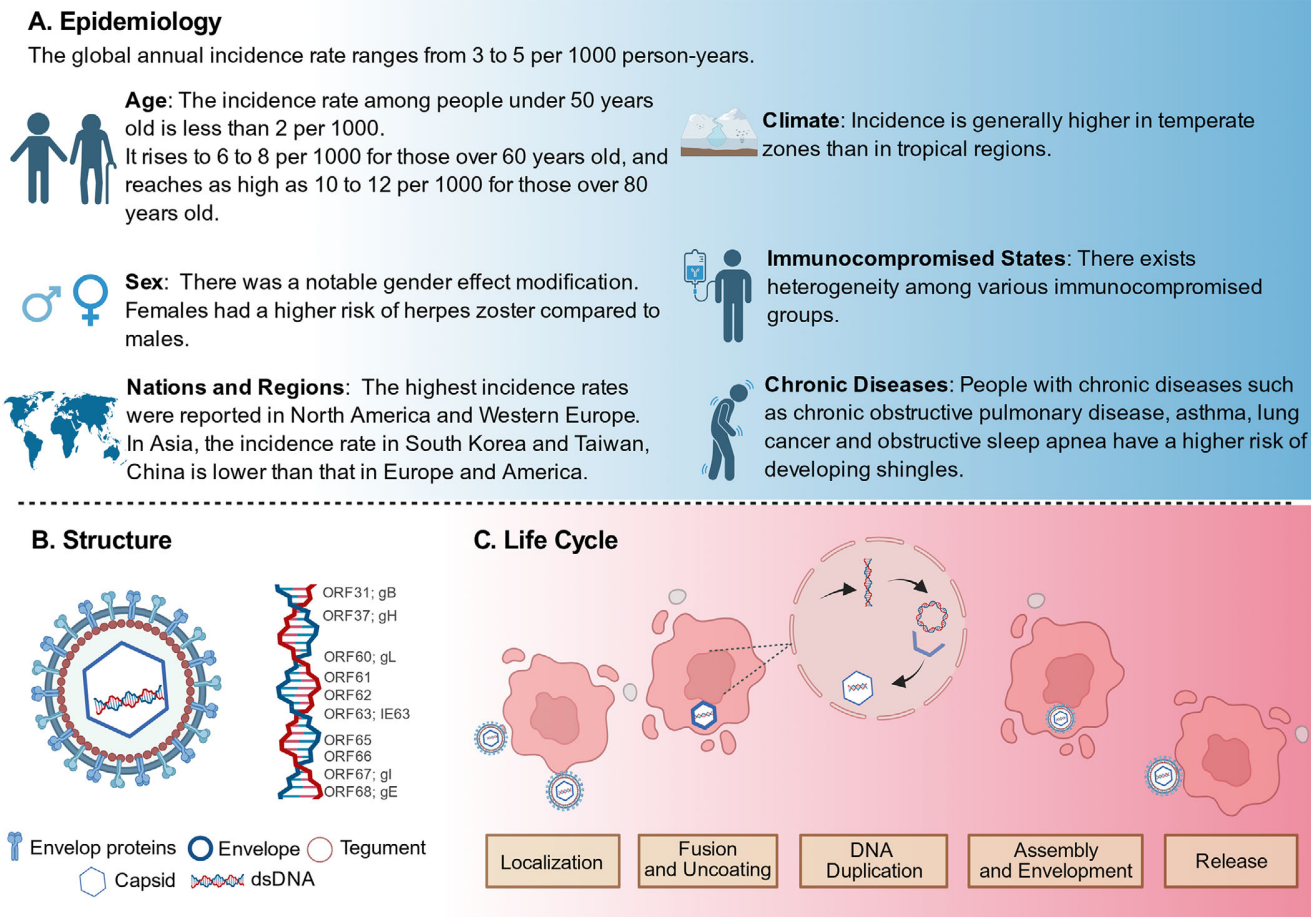
Epidemiology, structure, and life cycle of VZV. This figure provides an overview of VZV, including its epidemiological characteristics (A), virion structure (B), and replication life cycle (C). Together, these panels summarize key biological features that underpin VZV transmission, latency establishment, and reactivation. *Abbreviation*: VZV, varicella zoster virus.

Multiple factors influence the incidence of HZ, including advanced age [5], sex [5, 22], geographic location [22], climate [22], and immunosuppressive status [23] (Figure 1A).

#### 2.1.1 Age and Sex

Among these determinants, the age‐related decline in VZV‐specific cell‐mediated immunity, which typically accelerates after the age of 50 years, constitutes a critical risk factor [3] (Figure 1A). This process of immunosenescence drives a steep rise in the incidence, hospitalization, and case‐fatality rates of HZ with advancing age [3]. Consistent with this mechanism, global epidemiological data indicate an incidence rate of 5.23 to 10.9 per 1000 person‐years specifically in the population aged 50 years and older [5]. This age‐dependent pattern is further reflected in age‐standardized metrics: while the age‐standardized incidence rate (ASIR) and age‐standardized DALY rate (ASDR) exhibit a sharp decline in individuals under 20 years [5], both rates demonstrate a substantial increase in middle‐aged and elderly populations over the age of 50 years [5]. The incidence of HZ is consistently higher in women than in men [5, 22, 24]. This disparity is likely attributable to a combination of factors, including sex‐related differences in immune function, the longer life expectancy of women, and their greater propensity to seek medical care [25]. Supporting this observation, a meta‐analysis by Kawai et al. reported a summary adjusted relative risk of 1.31 for HZ in females compared with males [22].

#### 2.1.2 Regions, Nations, and Climate

Substantial geographical heterogeneity characterizes the global burden of HZ. Regionally, South Asia bears the highest incidence of VZV infection, followed by East Asia and Sub‐Saharan Africa [22, 26]. An analysis of 204 countries and territories in 2019 revealed a pronounced disparity in the ASIR and ASDR of VZV. Specifically, the ASDR varied by more than 39‐fold, ranging from 55.68 in Somalia to 1.36 in Slovenia [5]. Racial differences are also evident; for instance, Black individuals exhibit nearly half the risk of HZ compared with their White counterparts [22]. Additionally, the implementation of universal varicella vaccination in countries such as the United States and Canada has successfully reduced chickenpox incidence but has been associated with a concurrent rise in HZ incidence among older adults [22]. This shift may be attributable to reduced natural boosting of cell‐mediated immunity due to diminished wild‐type (WT) VZV circulation in the community. Furthermore, climatic conditions appear to influence HZ epidemiology. Incidence is generally higher in temperate zones than in tropical regions, where primary VZV infection often occurs later in life, resulting in a distinct epidemiological pattern for HZ [22] (Figure 1A).

#### 2.1.3 Immunocompromised States and Chronic Disease

Immunocompromised adults are at a significantly elevated risk of developing HZ compared with immunocompetent individuals [23]. In some younger immunocompromised adults, the HZ incidence may approach or even surpass that observed in adults over 50 years of age from the general population [23]. However, substantial heterogeneity exists across different immunocompromised subgroups. For example, the median cumulative incidence in hematopoietic stem cell transplant (HCT) recipients is 18.6%, whereas it remains below 10% in patients with hematological malignancies, solid tumors, or Human immunodeficiency virus HIV [23, 27]. The incidence among solid organ transplant (SOT) recipients varies widely [28, 29] (0–16%), influenced by the transplanted organ type. When expressed as incidence per 1000 person‐years, the rate in HCT patients (43 out of 1000) is nearly triple that in SOT or HIV patients [28, 29] (each 17 out of 1000). Additionally, autoimmune conditions such as rheumatoid arthritis [30] and systemic lupus erythematosus [31] are also associated with a significantly increased risk of HZ (Figure 1A). The risk of HZ is significantly elevated by comorbidities such as COPD [32, 33], asthma [32, 33], diabetes [34], and cancer [23, 35], with risk compounding in individuals with multiple conditions. Given this increased burden, targeted vaccination is recommended. High‐risk groups, including those with chronic diseases or immunosuppression, should initiate vaccination from the age of 50 years, with coverage expanded to all adults at the age of 64 years (Figure 1A).

### 2.2 Structure and Life Cycle

With a diameter ranging from approximately 150 to 200 nm, the virion has a spherical morphology and is structurally organized, from the exterior to the interior, into several distinct layers: the glycoprotein‐containing envelope, the nucleocapsid, and the tegument protein layer [36, 37] (Figure 1B). Notably, glycoproteins embedded in the viral envelope, function as key surface proteins that mediate viral recognition and attachment to host cells. To date, eight VZV glycoproteins have been identified. Among these, glycoprotein E (gE) [38], gB [39], gH [40], and gI [41] represent the major immunogenic proteins responsible for stimulating specific antibody production (Figure 1B). The gB/gH–gL complex plays a critical role in mediating both virus–cell and cell–cell membrane fusion, a process that ultimately results in the creation of multinucleated syncytia [41]. It is noteworthy that the function of gB is finely regulated. Its cytoplasmic domain contains an immunoreceptor tyrosine‐based inhibition motif (ITIM), and the phosphorylation of this motif is critical for preventing excessive fusion, thereby enabling efficient replication in the skin [41]. Concurrently, a furin cleavage site within its extracellular domain is also necessary for skin pathogenicity [41]. The gH–gL heterodimer activates the fusogenic function of gB, and specific regions within its extracellular domain, such as the N‐terminus of Domain I and the fusion loops in Domain III, have been demonstrated to be crucial for skin infection [42, 43]. In contrast to this core “fusion engine,” gE and its partner gI play more complex “navigation and regulation” roles [44]. gE is one of the most important and unique glycoproteins of VZV. Its large, nonconserved N‐terminal region (amino acids 1–187) is indispensable for viral replication in both T cells and the skin [44]. The formation of a heterodimer with gI is crucial for the maturation and membrane expression of gE, the incorporation of gI into virions, and the efficient cell‐to‐cell spread of the virus within the skin, although this interaction has a lesser impact on T cell infection [44, 45]. Furthermore, the interaction between gE and the host cellular protein insulin‐degrading enzyme is thought to contribute to skin infection but is dispensable for infection of T cells and ganglia [46]. The cytoplasmic domain of gE contains functional endocytosis and Golgi targeting motifs, with the latter playing a role during in vivo infection [46]. gI itself is essential for the proper trafficking of gE and for the secondary envelopment of virions; deletion of gI completely abrogates viral replication in both T cells and the skin [47]. Interestingly, however, in dorsal root ganglia, the absence of gI results in an aberrant chronic infection rather than the establishment of typical latency. This finding highlights the critical role of gE–gI interactions in preventing neuronal destruction and indicates distinct requirements for infection in neuronal cells compared with T cells and the skin [41]. In summary, the functions of these glycoproteins exhibit significant differences across various tissues, reflecting the finely tuned, tissue‐specific pathogenic mechanisms of VZV (Figure 1B). VZV initiates infection through gB/gH/gL‐mediated fusion, with its genome replicating in the nucleus under the regulation of viral tegument proteins like IE62 [41, 48, 49] (Figure 1C). Virion assembly involves nucleocapsid budding at the inner nuclear membrane and secondary envelopment at the trans‐Golgi network, with mature virus spreading primarily via cell–cell fusion and syncytia formation rather than free virion release [12, 50, 51] (Figure 1C).

### 2.3 Research Trends

**FIGURE 2:**
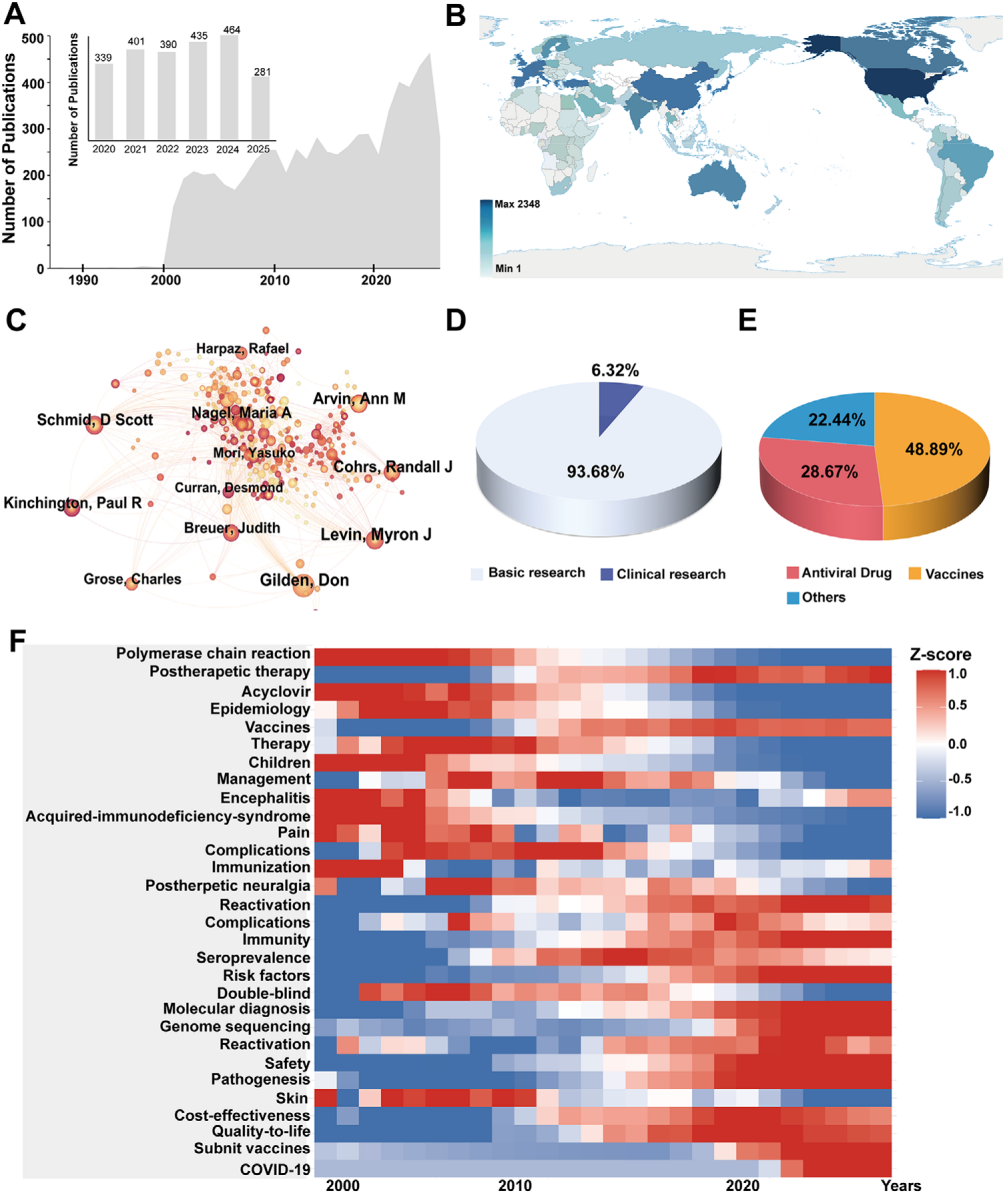
The research trends of VZV through bibliometric analysis. (A) Number of publications per year from 1990 to 2025. (B) Number of National Publications. The color intensity of each node represents the level of influence of each country in the field of VZV. (http://bzdt.ch.mnr.gov.cn/; Map approval number: GS(2016)1663) (C) Collaborative relationships among authors. Each author is represented by a different color, and the size of each line indicates the degree of collaboration. (D) Proportion of basic and clinical research in the study of VZV. The white section of the pie chart represents basic research, while the purple section represents clinical research. (E) The proportion of different types of studies in clinical research. The red section of the pie chart represents antiviral drug research, while the yellow section represents vaccine research. (F) The keyword trend map illustrates the change in frequency of keywords, with different colors representing varying levels of frequency over time.

To elucidate the evolving research landscape and current priorities in VZV research, we employed bibliometric analysis—a quantitative methodology for mapping scientific domains and identifying emerging trends [52, 53] (Figure S1). A systematic literature search was performed using the Web of Science Core Collection database with the predefined search query: (“Varicella zoster virus” OR “VZV”) AND (“Herpes zoster” OR “Shingles”). Following the exclusion of irrelevant publication types, a total of 7118 articles were retained for subsequent analysis. Analyses were carried out using established bibliometric tools, including CiteSpace, VOSviewer, and the R programming language (Figure S1). Based on our bibliometric analysis, we have derived the following preliminary conclusions. As shown in Figure 2A, the number of publications on VZV is substantial and has demonstrated consistent growth, reflecting its significance as a public health concern and sustained scientific interest. A notable peak occurred in 2020, with a subsequent sustained upward trend (Figure 2A). This surge coincides temporally with the global approval and widespread application of the RZV. Furthermore, analysis of national publication networks and author collaboration maps reveals that research in this field is led by top academic institutions and pharmaceutical companies in the Canada, United States, Europe, Japan, and China (Figure 2B,C). This suggests that the development of therapeutic agents and preventive measures, particularly vaccines, represents one of the most intensely invested and competitive directions in current global VZV research. Heatmap analysis of keywords indicates a clear shift in research themes (Figure 2F). Early studies focused predominantly on “children,” “epidemiology,” and treatment with “acyclovir,” representing the initial phase of defining clinical manifestations and foundational antiviral therapies (Figure 2F). The research focus subsequently shifted toward “postherpetic neuralgia,” “complications,” and “management,” addressing more complex and chronic clinical aspects and the need for more effective treatment strategies (Figure 2F). Recent high‐frequency keywords include “vaccine,” “immunity,” “cellular immunity,” “latent infection,” and “reactivation.” Additionally, “molecular diagnosis” and “genome sequencing” have emerged as frontier topics (Figure 2F). Finally, despite considerable achievements in clinical and preventive research, the translation of fundamental molecular discoveries into novel therapeutic interventions remains relatively underdeveloped (Figure 2D,E). In summary, while significant progress has been made in understanding the clinical manifestations, molecular pathogenesis, and prevention via vaccination, our bibliometric analysis also highlights a critical gap: the understanding of the core molecular mechanisms governing latent infection has not yet been sufficiently translated into transformative treatment strategies for HZ.

## 3 Clinical Features

**FIGURE 3:**
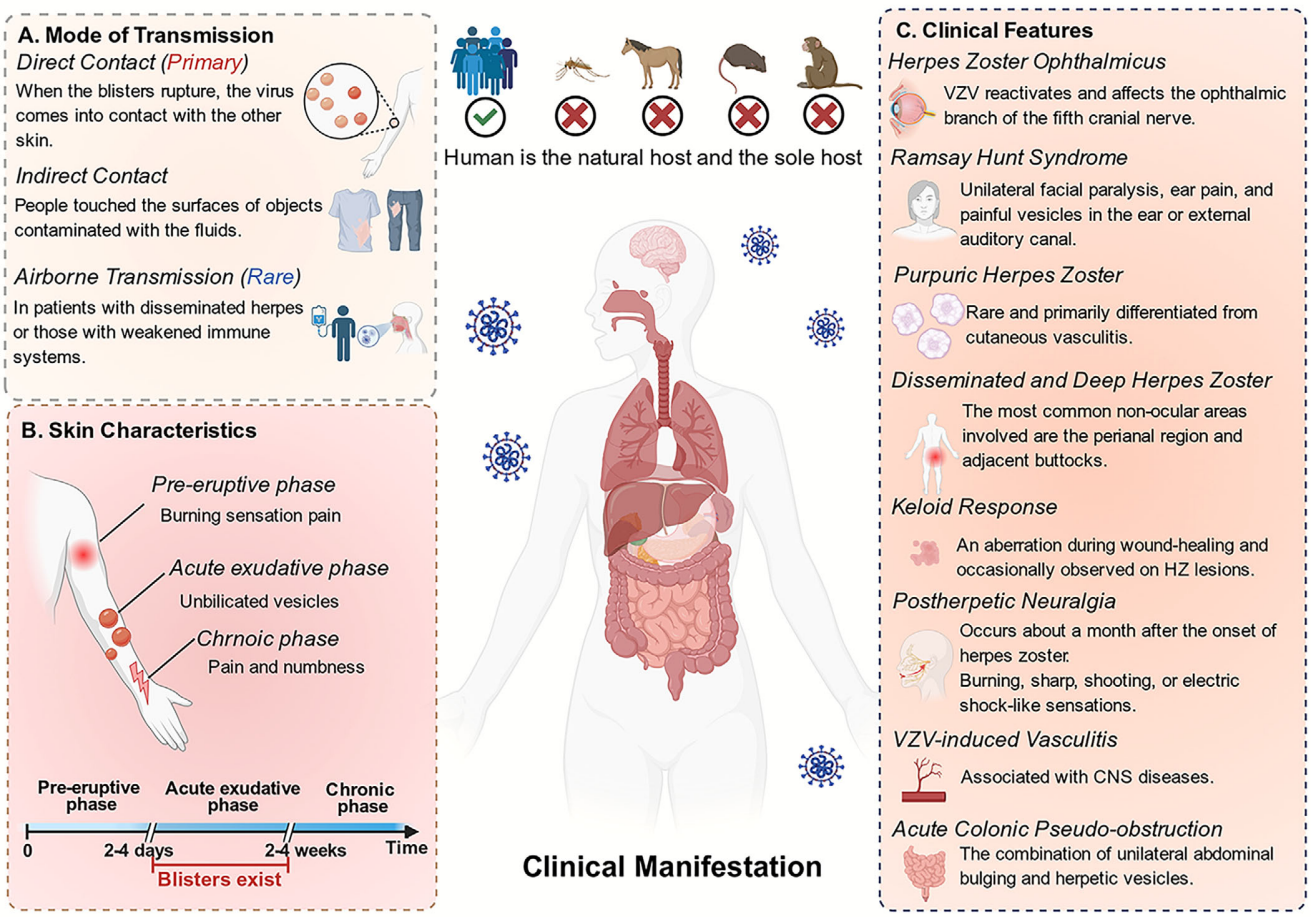
Clinical manifestations and transmission routes of VZV infection. This schematic illustrates (A) the major modes of VZV transmission, (B) characteristic cutaneous manifestations, and (C) the spectrum of clinical features associated with VZV infection. The figure highlights the progression from primary infection to clinically evident disease.

Susceptible individuals can get infected if they come into direct contact with the blister fluid of a person with varicella (Figure 3A). The virus can enter the body through tiny skin wounds or mucous membranes, causing an infection. The clinical presentation of HZ is characterized by a progression from localized dermatomal symptoms to potentially severe systemic complications (Figure 3B,C).

### 3.1 Cutaneous Manifestations

The cutaneous manifestations of HZ follow a characteristic triphasic evolution [54]: the prodromal phase presents with burning sensations, tingling, and pruritus within the affected dermatome. The acute eruptive phase is characterized by unilateral, dermatomal clusters of umbilicated vesicles on an erythematous base, which evolve through stages of pustulation and crusting; this phase is highly contagious and typically associated with intense pain. The chronic phase is defined by persistent sensory abnormalities, such as dysesthesias or paresthesias, in the healed dermatome after the rash has resolved (Figure 3B). Several distinct clinical patterns are recognized. Involvement of the ophthalmic division (V1) of the trigeminal nerve results in HZ ophthalmicus (HZO), presenting with vesicles on the forehead, tip of the nose, and periorbital region, and complicating 50–85% of cases with conjunctivitis, keratitis, uveitis, or retinitis [55]. Ramsay hunt syndrome (RHS) Promyelocytic leukemia, caused by VZV reactivation in the geniculate ganglion, is characterized by vesicles in the external auditory canal and pinna accompanied by ipsilateral facial palsy [56]. Immunocompromised individuals may develop disseminated cutaneous lesions or deep perianal ulcerations [57]. Zoster sine herpete presents with radicular pain in a dermatomal distribution without any accompanying rash [58]. Following rash resolution, some patients may develop keloids at the site of healed lesions as an isotopic response [59]. Other late cutaneous sequelae include the formation of pseudoherniations and cutaneous cysts [60, 61] (Figure 3C). Overall, based on the characteristic triphasic evolution and diverse clinical patterns, HZ presents with a spectrum of cutaneous manifestations and potential sequelae.

### 3.2 Neurological Manifestations

Pain is the core symptom, characterized by severe neuropathic pain in the acute phase. The most representative complication is PHN, defined as pain persisting for more than 4 weeks after rash healing. The clinical presentation of PHN includes burning or electric shock‐like pain and allodynia, which can persist for months to years [62]. Involvement of the central nervous system presents in diverse forms: vascular pathology is the most significant, where VZV infection of intracranial arteries leads to vasculitis, potentially causing ischemic or hemorrhagic stroke, aneurysm, or dissection. This can occur weeks to months after the rash, or even precede it without a rash prodrome [54]. The association between giant cell arteritis and VZV remains controversial; detection rates of VZV antigen in temporal artery biopsies vary widely (12–74%), and a causal relationship awaits confirmation [56, 63, 64, 65, 66]. Immunocompromised patients are susceptible to meningoencephalitis, presenting with headache, altered consciousness, and seizures, with an annual incidence of approximately 5.3 per million [58]. Spinal cord involvement can lead to segmental motor weakness and acute myelitis, manifesting as bilateral lower limb paralysis and sphincter dysfunction [67]. Cranial neuropathies extend beyond the classic facial nerve palsy, with ophthalmic division involvement of the trigeminal nerve potentially causing ophthalmoplegia. A rare complication is Guillain‐Barré syndrome (GBS), which carries a poor prognosis [68] (Figure 3C). In summary, the neurological manifestations of HZ range from acute neuropathic pain to diverse and severe complications affecting both the peripheral and central nervous systems.

### 3.3 Digestive System Manifestations

VZV can establish latency and subsequently reactivate within the enteric nervous system, leading to the formation of gastric and colonic ulcers, which clinically present as abdominal pain and gastrointestinal bleeding [69]. A rare complication is acute colonic pseudo‐obstruction, characterized by severe abdominal distension and ileus. Only 28 cases were reported between 1950 and 2008, with a mean patient age of 61 years and a male predominance of 76% [70]. Furthermore, rare cases of IgA vasculitis can be accompanied by severe gastrointestinal symptoms [71] (Figure 3C).

### 3.4 Urinary System Manifestations

HZ virus infection may also be associated with urinary system symptoms. Aseptic meningitis can be complicated by acute urinary retention, a condition termed meningitis‐retention syndrome. Patients present with meningeal signs such as fever, headache, and nuchal rigidity, while the bladder is initially characterized by an areflexic state [72]. This syndrome is considered a mild form of acute disseminated encephalomyelitis (Figure 3C).

## 4 Molecular Pathogenesis

**FIGURE 4:**
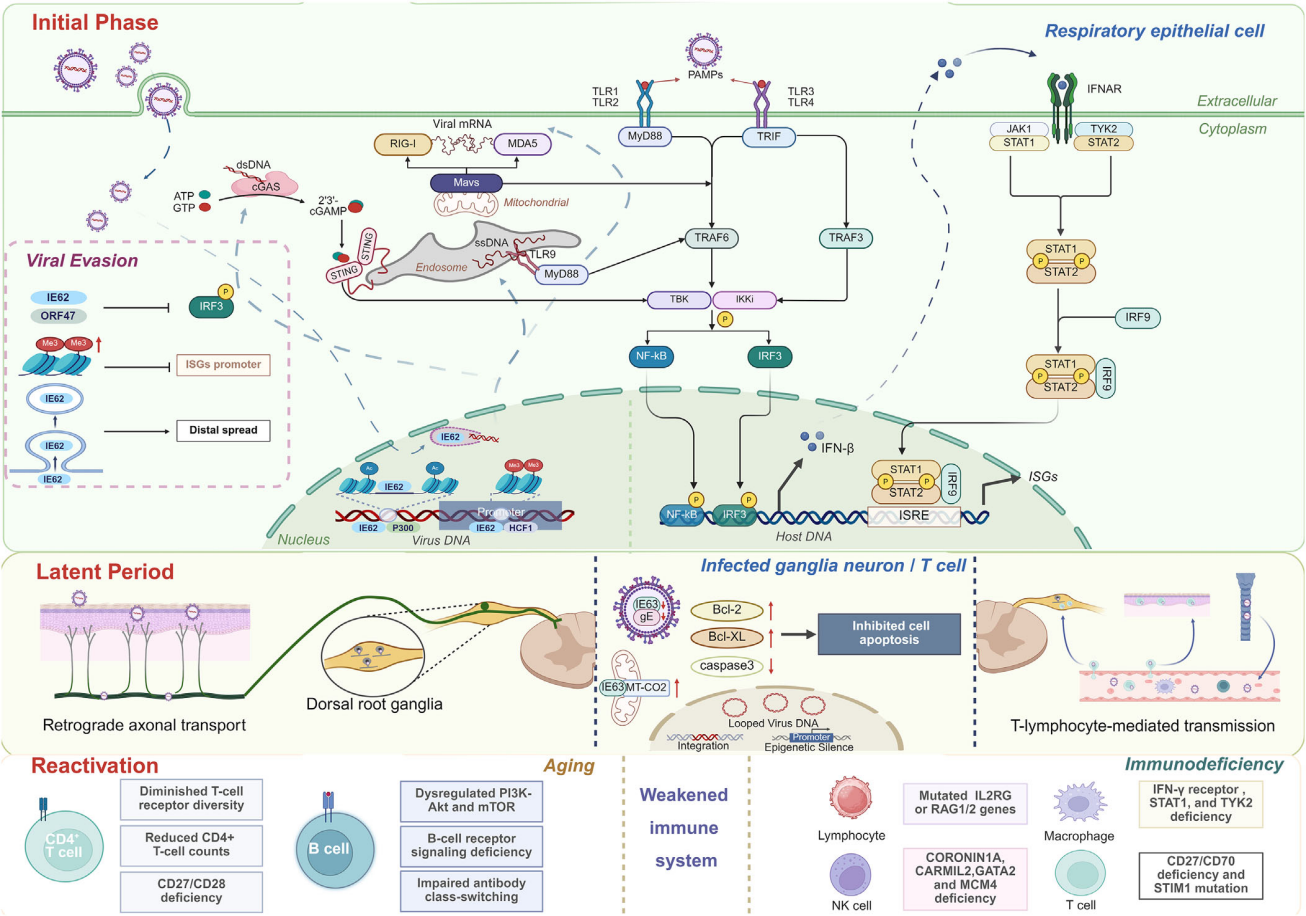
Host immune responses during different stages of VZV infection. This figure depicts the host immune responses elicited during distinct stages of VZV infection. Innate and adaptive immune mechanisms involved in viral recognition, control, and clearance are summarized across the course of viral invasion.

The pathogenesis of HZ primarily involves initial infection with the VZV, followed by viral latency and subsequent reactivation. However, the clinical manifestations are mainly driven by three key mechanisms: persistent viral replication during the reactivation phase, the host immune response triggered by the virus, and sequelae resulting from virus‐induced neuronal damage [73] (Figure 4).

### 4.1 Overview of the Infection Mechanism

#### 4.1.1 Initial Phase

VZV infection is initiated when the virus enters respiratory mucosal epithelial cells through engagement of surface receptors such as the mannose‐6‐phosphate receptor or myelin‐associated glycoprotein. Viral envelope glycoproteins gB, gH, and gL coordinate this process by mediating membrane fusion or endocytosis [74, 75]. Following entry, the viral envelope is shed and tegument proteins are released into the cytoplasm, among which IE62 serves as the principal transcriptional activator that initiates viral gene expression. First, it binds host p300 histone acetyltransferase to enhance histone acetylation [48, 76, 77], and second, it recruits host cell factor 1 and histone methyltransferase complexes to viral promoters, establishing transcriptionally active chromatin marked by H3K4me3 and H3K36me3 that drives expression of the VZV genome—a linear double‐stranded DNA (dsDNA) containing at least 71 Open reading frames (ORFs) that replicates and assembles in the nucleus [78, 79, 80].

The innate immune response to VZV infection is initiated by the recognition of viral components by host pattern recognition receptors (PRRs). These include cell surface receptors such as Toll‐like receptor (TLR) double‐stranded DNA 2, TLR1/2 complexes, and TLR9, which specifically senses VZV dsTIR‐domain‐containing adapter‐inducing interferon‐βDNA, as well as intracellular sensors including NOD‐like receptor family Pyrin domain containing 3 (NLRP3), Melanoma differentiation‐associated protein 5(MDA5), Retinoic inducible gene I(RIG‐I), RNA Polymerase III, and cyclic GMP‐AMP synthase (cGAS)Tumor necrosis factor‐alpha [81, 82, 83].

Activation of PRRs triggers robust type I interferon (IFN) responses through parallel signaling pathways. TLR3 and TLR4 recruit the adaptor protein TIR‐domain‐containing adapter‐inducing interferon (TRIF), leading to the activation of TNF receptor‐associated factor (TRAF)3 and the TANK‐binding kinase (TBK)1/IκB kinase ε (IKKε), which subsequently phosphorylate IFN regulatory factor (IRF)3 and promote its nuclear translocation [84, 85]. Similarly, RIG‐I and MDA5 signal through the mitochondrial adaptor Mitochondrial antiviral‐signaling (MAVS) proteins to activate TBK1/IKKε and induce IRF3 phosphorylation [84, 85]. Secreted IFN‐α/β then engages the Janus kinase (JAK)–Signal transducer and activator of transcription (STAT) pathway, activating JAK1 and Tyk2 to phosphorylate STAT1 and STAT2. Together with IRF9, these factors form the ISGF3 complex, which induces a broad spectrum of IFN‐stimulated genes (ISGs), including protein kinase R, 2'‐5'‐oligoadenylate synthetase (OAS)/ribonuclease L (RNase L),Myxovirus resistance (Mx) proteins, and Interferon‐induced protein with tetratricopeptide repeats (IFIT) family members, thereby establishing an antiviral state [84, 85].

To counteract these defenses, VZV has evolved multiple, layered immune evasion strategies. The major immediate‐early protein IE62 inhibits IRF3 phosphorylation and nuclear import, thereby suppressing IFN‐β production, while the viral kinases ORF47 and ORF66 further impair IRF3 and STAT1 activation, respectively [48, 76, 77]. In addition, IE63 interferes with eIF2α phosphorylation and antagonizes IFN‐α‐mediated antiviral functions [48]. Beyond direct signaling inhibition, VZV induces sustained transcriptional silencing of host antiviral genes through promoter hypermethylation. For example, hypermethylation of IRF9 disrupts JAK–STAT signaling, Tumor necrosis factor (TNF)Cyclin‐dependent kinase‐α promoter methylation attenuates proinflammatory cytokine production, and TLR3 hypermethylation compromises viral RNA sensing [86, 87, 88]. Methylation of the Cyclin‐dependent kinase （CDK ）2 promoter may further arrest the host cell cycle, creating a cellular environment favorable for viral replication [88].

Additional immune evasion mechanisms include suppression of NF‐κB activation via inhibition of Inhibitor of kappa B alpha (IκBα) degradation [84], modulation of apoptosis and autophagy pathways [89, 90, 91], and the packaging of IE62 into small extracellular vesicles to mediate distal immunosuppression [92]. Moreover, VZV directly targets immune effector cells by infecting and functionally impairing natural killer cells, thereby reducing cytotoxic activity and cytokine production [93]. The virus can also hijack IFN‐γ signaling through glycoprotein C to upregulate Intercellular adhesion molecule (ICAM)‐1 expression on infected keratinocytes, enhancing T cell adhesion and facilitating viral dissemination within the skin [94] (Figure 4).

Collectively, these findings indicate that during the initial phase of infection, VZV not only establishes productive replication within host cells but also actively subverts innate immune defenses through coordinated molecular, epigenetic, and cellular processes.

#### 4.1.2 Latent Period

VZV achieves latency in sensory ganglia via two complementary routes: retrograde axonal transport and T lymphocyte‐associated viremia. Upon reaching neuronal cell bodies, the virus shifts from a lytic to a latent program, characterized by limited viral gene expression, suppression of apoptosis, and epigenetic repression of the viral genome. This intricate strategy ensures the lifelong persistence of VZV, creating the reservoir for subsequent reactivation as HZ (Figure 4).

Following initial replication in the skin, VZV invades peripheral sensory nerve endings and exploits retrograde axonal transport, primarily via the dynein motor complex, to reach neuronal cell bodies in sensory ganglia such as the dorsal root ganglia [4]. In contrast to the lytic infection observed in epithelial cells, neuronal infection preserves cellular integrity and basal protein synthesis. This transition is accompanied by nuclear sequestration of viral transactivators and reduced envelope glycoprotein production, hallmarks of latency establishment [91]. Consistently, infected neurons display decreased expression of IE63 and gE, along with reduced apoptotic markers, including caspase‐3 [95]. Concurrent upregulation of antiapoptotic proteins Bcl‐2 and Bcl‐XL, together with colocalization of IE63 and the mitochondrial protein MT‐CO2, indicates active viral suppression of neuronal apoptosis [96]. Mechanistically, this state is reinforced by ORF63 relocalization, ORF12‐mediated modulation of ERK signaling, and ORF66‐dependent inhibition of apoptosis, collectively promoting long‐term persistence [89, 90, 91].

Once latency is established, the viral genome persists predominantly as a circular episome within the neuronal nucleus, maintained in a quiescent state through active epigenetic regulation. Chromatin organization plays a pivotal role, with promoters of key genes like ORF62 and ORF63 associated with histone H3K9 acetylation—a euchromatic mark that preserves transcriptional potential without prompting reactivation [97]. Further computational analyses reveal the presence of CTCF binding sites located in the viral latency‐associated transcript (VLT) region. The chromatin architectural protein CTCF is postulated to organize the viral genome into loop structures, thereby repressing lytic genes (e.g., ORF14, ORF36) while potentially coordinating the coregulation of VLT and ORF63, serving a dual function in epigenetic silencing and latent transcript coordination [98, 99, 100, 101]. VLT itself is a polyadenylated RNA antisense to ORF61, and its low‐level expression contributes to viral quiescence by suppressing ORF61 transcription [102]. The antiapoptotic function of IE63 (encoded by ORF63) remains critical for latency maintenance, preventing neuronal death and aiding immune evasion by delaying host cell destruction [103, 104]. In vivo evidence from the severe combined immunodeficiency (SCID‐hu) model corroborates that direct VZV inoculation leads to neuronal replication followed by a transition to latency, with the viral genome maintained at approximately two to nine copies per cell in about 4% of neurons and ORF63 representing a predominant transcript [105, 106, 107] (Figure 4).

Complementing direct neuronal spread, VZV utilizes a systemic hematogenous pathway via T‐lymphocytes to disseminate and establish latency in geographically distant ganglia. The virus is highly specific for T cells, with a marked preference for CLA‐ and CCR4‐expressing memory CD4+ T cells [108, 109]. Following initial replication in the respiratory mucosa, VZV disseminates to tonsillar and other lymphoid tissues, where it infects T cells to establish a cell‐associated viremia. Infected T lymphocytes then serve as vehicles, transporting the virus via the bloodstream not only to the skin but also, critically, to remote sensory ganglia, including dorsal root and cranial nerve ganglia, thereby seeding latent infection at these distant sites [108, 109]. VZV enhances this process by promoting T‐cell survival; for instance, it induces STAT3 activation and subsequent survivin expression, which protects infected T cells from apoptosis and ensures efficient viral delivery to target tissues [110]. This T‐cell‐mediated metastasis provides a plausible explanation for how VZV can establish latency in ganglia far removed from the initial site of infection. The occurrence of WT VZV zoster in vaccinated children without a history of clinical varicella supports this model, thereby suggesting that the initial ganglionic infection occurs via a blood‐borne route [105]. Furthermore, in vivo models, including the SCID‐hu system, directly demonstrate that VZV‐infected human tonsillar T cells can transfer the virus to dorsal root ganglia xenografts and establish latent infection, confirming that VZV co‐opts immune cell trafficking for systemic dissemination and the establishment of a latent reservoir within disparate ganglia [108, 109] (Figure 4).

#### 4.1.3 Reactivation

Among the numerous factors contributing to VZV reactivation, advanced age and immunodeficient states are the most prominent. The age‐related waning of cell‐mediated immunity is a key driver of this process. It is marked by a reduction in T‐cell receptor diversity, declining CD4+ T‐cell numbers, and decreased CD28/CD27 expression, which together compromise IL‐2 production and T‐cell proliferative capacity [111, 112]. This immunosenescence phenomenon further involves dysregulated PI3K–Akt and mTOR signaling pathways, alongside defects in B‐cell receptor signaling and antibody class‐switching mechanisms [111, 112]. Notably, VZV infection activates multiple innate immune pathways in senescent cells, including TLR, NF‐κB, and p38 MAPK signaling, establishing a dynamic equilibrium between viral replication efficiency and host immune status [103, 111, 112, 113]. At the tissue level, VZV‐specific CD4+ central memory T‐cells and Foxp3+ regulatory T‐cells form an immunosuppressive network in the cutaneous microenvironment through PD‐1 upregulation and suppression of IFN‐γ/IL‐2 secretion, creating favorable conditions for viral reactivation [113]. Beyond age‐related factors, primary immunodeficiencies represent another crucial risk dimension. Patients with SCID carrying mutations in IL2RG or RAG1/2 genes develop lymphocyte maturation defects that predispose to disseminated infections [114, 115]. Deficiencies in CORONIN1A and CARMIL2 primarily compromise T/NK cell function [93], while GATA2 and MCM4 defects lead to quantitative or functional NK cell abnormalities [116, 117, 118]. Specific immunodeficiencies, including IFN‐γ receptor defects, loss‐of‐function STAT1 mutations, and TYK2 deficiencies, compromise macrophage activity and predispose individuals to severe infections [119], whereas CD27/CD70 deficiencies and STIM1 mutations disrupt T‐cell activation and calcium signaling, respectively [120, 121]. In acquired immunodeficiency contexts, malignancies, organ transplantation, and HIV/AIDS‐induced CD4+ T‐cell exhaustion significantly increase dissemination risks, while novel immunomodulators like JAK inhibitors promote central nervous system complications by interfering with IFN signaling pathways [121, 122, 123] (Figure 4).

### 4.2 Complications Driven by Continuous Viral Replication

The reactivation of VZV and its subsequent failure to be contained by the host immune system can lead to a broad spectrum of complications, extending far beyond the initial dermatome. These conditions are unified by a common pathophysiology: continuous viral replication that directly damages tissues and triggers persistent inflammation. The resulting clinical manifestations are diverse, ranging from localized cutaneous spread to severe neurological and systemic dissemination. The following sections detail several representative examples of such complications, including, but not limited to, severe cutaneous manifestations, meningoencephalitis, disseminated HZ (DHZ), and VZV‐induced vasculitis (Figure S2).

#### 4.2.1 Cutaneous Manifestations

Upon reactivation, VZV initiates extensive replication within epidermal keratinocytes. Key immediate‐early proteins such as IE62 efficiently suppress the host transcriptional machinery, thereby facilitating large‐scale viral replication [48, 49]. To overcome innate immune barriers of the skin, the virus employs multiple strategies: proteins including IE62, ORF47, and IE63 inhibit IRF3 and its downstream signaling, while the ORF66 kinase impedes STAT1 activation. These actions collectively attenuate the host's critical antiviral IFN pathway [51, 124, 125, 126]. Concurrently, VZV suppresses NF‐κB activation, thereby limiting the production of proinflammatory cytokines [127]. These immune evasion mechanisms facilitate robust viral proliferation, which correlates clinically with a relatively weak local immune response in early infection. The formation of pathognomonic multinucleated giant cells results from the fusion of infected cells with adjacent healthy cells, mediated by the viral glycoproteins gB and gH–gL expressed on their membranes—a process often accompanied by intranuclear eosinophilic inclusions [43]. This process is tightly regulated by the ITIM within the cytoplasmic domain of gB to prevent excessive fusion [128]. Such direct cell–cell fusion allows the virus to evade extracellular exposure and represents a key mechanism for efficient viral dissemination. Pathologically, this accounts for the spongiotic edema and syncytia formation within the epidermis—a characteristic histopathological hallmark of HZ. Meanwhile, viral replication, possibly through viral protease activity, may disrupt intercellular desmosomal junctions (e.g., E‐cadherin), inducing acantholysis. These processes collectively lead to ballooning degeneration of keratinocytes—featuring cell swelling, rounding, and eventual lysis. Furthermore, to sustain its replication niche, VZV activates the STAT3 signaling pathway, upregulating antiapoptotic proteins such as survivin, thereby delaying infected‐cell death [110]. This mechanism provides a temporal window for clinical lesion progression and substantial viral shedding. The viral ORF61 protein promotes the disassembly of Promyelocytic leukemia (PML) nuclear bodies, ensuring efficient nucleocytoplasmic release and virion assembly, which underlies the high titer of infectious virus within vesicles [129, 130]. Viral replication within keratinocytes releases viral pathogen‐associated molecular patterns, which are sensed by host PRRs such as cGAS–STING, activating downstream signaling pathways. Activated keratinocytes shift to a proinflammatory phenotype, secreting abundant chemokines (e.g., CXCL8/IL‐8, CXCL10) and proinflammatory cytokines (e.g., IL‐1β, IL‐6) [131]. These signals potently recruit neutrophils and lymphocytes into the epidermis. Ultimately, intraepidermal cavities form, filled with serous fluid, inflammatory cells, cellular debris, and high‐titer viral particles, corresponding to the clinically observed vesicles (Figure S2).

#### 4.2.2 VZV Encephalitis/Meningitis

The hallmark clinical features of VZV encephalitis/meningitis include acute headache, fever, nuchal rigidity, and photophobia in meningitis reflects the initial inflammatory assault on the meninges, driven by ongoing viral activity and the local release of key inflammatory mediators such as IL‐6 and TNF‐α [132, 133, 134]. Progression to encephalitis, marked by encephalopathy, seizures, and focal neurological deficits like hemiparesis or aphasia, signifies parenchymal invasion and persistent viral replication within the CNS. These symptoms are not merely a consequence of transient infection but are predominantly mediated by a robust immunopathological cascade sustained by continuous viral antigenic stimulation. A critical molecular determinant of this process is VZV's tropism for cerebral vasculature, leading to a granulomatous vasculitis [135, 136]. The persistent presence of replicating virus and viral antigens within the vessel wall initiates and maintains a delayed‐type hypersensitivity response, characterized by CD4+ T‐cell and macrophage infiltration, formation of granulomas, and resultant vascular inflammation, stenosis, and occlusion. This virus‐driven vasculopathy is the primary pathophysiological substrate for the ischemic strokes and transient ischemic attacks that clinically manifest as acute focal deficits [136]. The sustained viral replication is facilitated by sophisticated immune evasion strategies that enable VZV to persist in the immunologically privileged CNS environment. A key mechanism involves the viral ORF9 protein, which functions as a potent antagonist of the cytosolic DNA sensor cGAS. By binding to cGAS and inhibiting the synthesis of the second messenger cGAMP, ORF9 effectively blunts the downstream STING‐dependent production of type I IFNs, thereby fostering conditions permissive to extended viral replication [137, 138]. The consequent continuous viral activity triggers chronic activation of glial cells and sustained recruitment of peripheral immune cells, coordinated by a pronounced chemokine and cytokine milieu including CXCL10, CCL2, and CCL5 [139, 140]. This persistent neuroinflammatory state contributes to the disruption of the blood–brain barrier through cytokine‐induced upregulation and activation of matrix metalloproteinases (MMPs), which proteolytically degrade tight junction proteins and basement membrane components [141]. The long‐term cognitive sequelae observed in survivors, such as impairments in memory and executive function, are the functional correlate of this sustained viral presence and the associated chronic neuroinflammatory state [142, 143]. In summary, the clinical spectrum of VZV‐related CNS infections is fundamentally driven by persistent viral replication, which initiates and maintains an immunopathological process that results in both acute neurological deficits and long‐term sequelae (Figure S2).

#### 4.2.3 Disseminated HZ

DHZ represents a severe manifestation of VZV reactivation, fundamentally arising from severely impaired cell‐mediated immunity that fails to confine viral replication to the initial sensory ganglion and its corresponding dermatome [144, 145]. In immunocompetent hosts, VZV‐specific CD8+ cytotoxic and CD4+ helper T cells work in concert to recognize and clear infected cells, effectively curbing viral replication [145]. In contrast, under conditions of cellular immune deficiency, the virus evades local containment, enters the bloodstream, and initiates viremia. Through hematogenous dissemination, the virus reaches and replicates in distant cutaneous areas, a process clinically defined by the presence of over 20 vesicular lesions outside the primary and immediately surrounding dermatomes [145]. In severe presentations, the exanthem may become disseminated, taking on a varicelliform appearance that closely resembles primary chickenpox, and is characterized by the simultaneous presence of lesions in all stages of development.

DHZ occurs almost exclusively in individuals with profound cellular immune compromise. Patients considered at high risk include those with hematologic malignancies (e.g., lymphoma, leukemia) [145], solid organ or HCT recipients on long‐term immunosuppression, individuals with advanced HIV infection (particularly with CD4+ counts <200/µL) [146], and those receiving potent immunosuppressive therapies. Notably, this includes high‐dose corticosteroids, TNF‐α inhibitors, and JAK inhibitors such as tofacitinib and baricitinib. The latter agents directly impede crucial cytokine signaling pathways (e.g., IL‐2, IL‐7, IL‐15) essential for T‐cell activation and proliferation, thereby substantially compromising antiviral immunity [147].

The gravest consequence of DHZ is visceral involvement, resulting from hematogenous dissemination and subsequent viral replication in internal organs, leading to significant morbidity and mortality. Major complications include: VZV pneumonia, often presenting as interstitial pneumonia that can rapidly progress to ARDS and carries a high case‐fatality rate [148]; VZV hepatitis, typically characterized by asymptomatic transaminase elevation but rarely culminating in fulminant hepatic failure [149]; and VZV encephalitis, caused by hematogenous spread of the virus to the brain parenchyma, which can manifest as altered mental status and seizures [149] (Figure S2).

#### 4.2.4 VZV‐Induced Vasculitis

The pathogenesis of VZV vasculopathy originates from the reactivation of the virus in sensory ganglia and its subsequent anterograde transport to the arterial adventitia, to which it is conveyed via axons to innervating nerve terminals [150]. Subsequent viral replication initiates a robust inflammatory response. The early phase is marked by neutrophilic infiltration, which later evolves into a persistent inflammatory milieu dominated by CD4+ and CD8+ T cells and CD68+ macrophages within the arterial wall [151]. These infiltrating immune cells, together with VZV‐infected vascular cells, secrete proinflammatory cytokines such as IL‐6 and IL‐8, and MMPs (e.g., MMP‐2), which perpetuate inflammation and mediate vascular injury [132].

Pathological examination of affected arteries reveals three key features: disruption of the internal elastic lamina, apoptosis of medial smooth muscle cells leading to loss of wall integrity, and intimal hyperplasia driven by the accumulation of α‐smooth muscle actin‐positive myofibroblasts, which can culminate in luminal stenosis and ischemic infarction [150]. The production of activated MMPs by both infected cells and neutrophils contributes to the degradation of the extracellular matrix, predisposing to vessel wall weakening, aneurysm formation, and potential rupture [152]. Furthermore, recent mechanistic studies demonstrate that VZV infection suppresses the surface expression of Major histocompatibility complex class (MHC)‐I and programmed death‐ligand 1 (PD‐L1) on human vascular endothelial cells in vitro [153]. This dual downregulation is postulated to facilitate viral persistence by impairing antigen presentation to cytotoxic T cells and by disrupting PD‐1/PD‐L1‐mediated immunoinhibitory signaling, thereby enabling the unchecked, chronic inflammation that characterizes this vasculopathy [153] (Figure S2).

### 4.3 Complications Driven by Inflammatory Responses

Beyond complications arising from continuous viral replication, a distinct subset of HZ sequelae is primarily mediated by the host's own dysregulated and often persistent inflammatory and immune responses to the virus. This immunopathology is frequently characterized by T‐cell‐dominated inflammation, cytokine release, and resultant structural injury in affected nerves and organs. The following sections illustrate this paradigm with key examples, including, but not limited to, the vision‐threatening ocular inflammation in HZO and the severe cranial neuritis characterizing RHS (Figure S2).

#### 4.3.1 HZO With Deep Keratitis/Uveitis

The pathogenesis of HZO begins with the reactivation of the latent VZV within the sensory neurons of the ophthalmic division (V1) of the trigeminal ganglion, typically occurring during periods of declined cell‐mediated immunity. The virus subsequently undergoes anterograde transport along the nerve axons, disseminating to the innervated skin and ocular tissues, where it causes direct infection and cytopathic damage [154]. This process manifests clinically in the acute phase. Cutaneous presentation includes a unilateral, dermatomal vesicular rash, and erythema. Involvement of the nasociliary branch, signaled by vesicles on the nasal tip (Hutchinson's sign), is a critical clinical indicator that strongly predicts a significantly elevated risk of ocular involvement [155]. Ocular manifestations resulting from direct viral replication encompass conjunctivitis, epithelial keratitis (characterized by elevated, branching pseudodendrites without terminal bulbs), and scleritis [156]. However, the most vision‐threatening complications of HZO arise from a chronic, immune‐mediated pathological process, illustrating a classic biphasic disease pattern. Following the acute infection, persistent VZV antigens within the corneal stroma and uveal tissues trigger a dominant T‐cell‐driven chronic inflammatory response [156, 157]. This sustained immunologic attack underlies a spectrum of severe sequelae: stromal keratitis and endotheliitis can lead to corneal opacity and edema; anterior uveitis (iritis/iridocyclitis) may cause iris atrophy, posterior synechiae, and complicated cataracts; neurotrophic keratopathy, stemming from combined viral and inflammatory damage to corneal nerves, results in corneal anesthesia, persistent epithelial defects, ulceration, and potential perforation [156, 158]. Furthermore, immune cell infiltration of the trabecular meshwork (trabeculitis) impairs aqueous humor outflow, leading to secondary glaucoma [157] (Figure S2).

#### 4.3.2 Severe Neuritis: RHS

HZ oticus, known clinically as RHS, is a serious cranial polyneuropathy that follows the reactivation of VZV [159]. The pathogenesis begins with the virus establishing latency within sensory neurons of the geniculate ganglion. Following a decline in host immunity, due to factors such as advanced age, stress, fatigue, or immunosuppression, the latent virus can reactivate [160]. The core mechanism of RHS involves an intense, focal immune‐inflammatory response triggered by viral replication. The robust infiltration of T lymphocytes and macrophages into the geniculate ganglion and facial nerve, along with a substantial release of proinflammatory cytokines following viral antigen stimulation, together precipitate a severe viral ganglionitis. The “inflammatory storm” within the confined bony space of the facial canal induces catastrophic edema of the neural tissues. Due to the limited space within the facial canal, this inflammatory edema causes significant compression of the nerve against the unyielding bony walls, ultimately resulting in an inflammation‐induced entrapment neuropathy. The consequent compromise of the microvascular circulation leads to neural ischemia, demyelination, and, in severe cases, axonal degeneration. This sequence of immunomediated injury constitutes the critical pathological basis for the frequently severe facial paralysis and poorer prognosis observed in RHS patients. This immune‐mediated neural damage directly dictates the clinical presentation of RHS. The classic triad of symptoms, acute unilateral facial palsy, intense otalgia, and herpetic vesicular eruptions in the auricular region, stems directly from the combined effects of viral cytopathy and the host inflammatory response. Severe otalgia originates from direct inflammatory injury to the sensory ganglion, while inflammatory edema and compression of the motor fibers of the facial nerve result in lower motor neuron‐type facial palsy, manifesting as facial asymmetry, incomplete eye closure, and drooping of the mouth. Furthermore, the complex functional anatomy of the facial nerve and its close proximity to the vestibulocochlear nerve (CN VIII) facilitate the spread of inflammation, generating symptoms beyond the classic triad. For instance, extension of inflammation to the chorda tympani nerve causes taste disturbances, while involvement of CN VIII leads to sensorineural hearing loss, tinnitus, and vertigo. Therefore, the entire clinical spectrum of RHS is initiated by VZV reactivation and is predominantly driven and amplified by the subsequent adaptive immune response and the resulting inflammatory nerve injury [161] (Figure S2).

### 4.4 Complications Resulting From Sequelae of Nerve Damage

A distinct category of HZ complications arises as sequelae of neuronal injury from the acute phase, rather than from active virus. The pathophysiology involves irreversible damage like neuronal loss and maladaptive plasticity, leading to chronic deficits such as postherpetic neuralgia and segmental motor paresis (Figure S2).

#### 4.4.1 PHN

PHN is a refractory neuropathic pain condition persisting for ≥3 months after acute HZ (AHZ) rash healing [162], characterized by constant burning/stabbing pain, allodynia, and severe pruritus [163], arising from altered peripheral and central sensory processing [164]. The pathogenesis of PHN is a multifaceted process initiated by the reactivation of the VZV, which causes direct neuronal damage and triggers a cascade of peripheral and central nervous system adaptations.

The process begins with peripheral sensitization, where nerve injury incites a local neurogenic inflammation and the infiltration of immune cells, such as macrophages, into the dorsal root ganglion (DRG). These cells release inflammatory mediators and chemokines (e.g., CCL2) that sensitize peripheral nociceptors, lowering their activation threshold [165, 166]. Concurrently, damaged neurons undergo an ion channel reorganization, characterized by the upregulated expression and altered function of voltage‐gated sodium channels (e.g., NaV1.8). This leads to neuronal membrane instability and spontaneous ectopic discharges, resulting in persistent pain signaling to the central nervous system even in the absence of external stimuli [167, 168].

This persistent afferent barrage drives central sensitization, a state of hyperexcitability in spinal cord pain pathways. This is mediated by several mechanisms: first, sensitized C fibers release excessive glutamate, which overactivates AMPA and NMDA receptors on spinal dorsal horn neurons. Sustained NMDA receptor activation causes significant Ca^2+^ influx, triggering intracellular cascades that long‐lastingly enhance synaptic efficacy [169, 170, 171]; second, there is a diminution of inhibitory control, as VZV‐induced damage can lead to the apoptosis or dysfunction of GABAergic and glycinergic interneurons, reducing the release of these inhibitory neurotransmitters and creating a state of spinal disinhibition [96, 162, 172]; and third, neuroimmune activation occurs, where spinal microglia and astrocytes become activated. Microglia are early responders, while astrocyte activation is closely linked to the long‐term maintenance of pain. These activated glial cells release proinflammatory cytokines, neurotrophic factors, and engage in signaling pathways (e.g., CXCL12/CXCR4), interacting with neurons to amplify and sustain central sensitization [173, 174, 175]. Furthermore, maladaptive structural reorganization occurs within the spinal cord, whereby non‐nociceptive, low‐threshold Aβ fibers sprout from deeper laminae (III–IV) into the pain‐processing superficial laminae (I–II), forming novel synapses. This aberrant rewiring underlies the phenomenon of tactile allodynia, where innocuous stimuli are perceived as pain [176, 177]. In some patients, severe nerve damage leads to deafferentation, a loss of sensory input that causes second‐order neurons to become abnormally hyperexcitable, generating spontaneous pain [178]. Contributing to this spontaneous and paroxysmal pain is the ectopic pacemaker hypothesis, which posits that sites of nerve injury, particularly the DRG, become generators of spontaneous pain signals independent of peripheral input [179, 180].

In summary, PHN arises from a complex network of interdependent mechanisms, including peripheral and central sensitization, neuroimmune activation, structural reorganization, deafferentation, and ectopic pacemaker activity, all initiated by VZV reactivation, which collectively underpin the refractory and complex nature of this chronic pain condition (Figure S2).

#### 4.4.2 Segmental Motor Palsy

Despite VZV's well‐known primary tropism for sensory nerves, its reactivation can propagate to the motor system, leading to segmental zoster paresis [181]. This complication manifests as focal lower motor neuron weakness in myotomes matching the affected dermatomes and is thought to occur through direct viral spread and inflammation from sensory ganglia to the anterior horn of the spinal cord [182].

An intense sensory ganglionitis, triggered by the epigenetic dysregulation of VZV, creates a high concentration of inflammatory mediators and viral particles. These can disseminate directly to the anatomically adjacent anterior gray matter of the spinal cord via intraneural or synaptic pathways. This results in damage to the alpha motor neurons located in the anterior horn, leading to their apoptosis or necrosis. The final outcome is a focal viral poliomyelitis, causing denervation and Wallerian degeneration of the muscle fibers innervated by the affected motor neurons. The muscle weakness typically develops within days to weeks after the onset of the rash, and its clinical presentation depends on the involved spinal segment. Limb paralysis is most common; cervical segment (C5–T1) involvement can lead to upper limb weakness (e.g., deltoid paresis, wrist drop) [183], while lumbosacral segment (L1–S4) involvement can cause lower limb weakness (e.g., foot drop). Diaphragmatic paralysis, though rare, is life‐threatening and results from viral invasion of the C3–C5 segments damaging the phrenic nerve, which can lead to respiratory difficulty. Thoracic segment (T6–T12) involvement may cause abdominal muscle weakness, resulting in abdominal bulging and the formation of a zoster pseudothermiation. Sacral zoster (S2–S4), by injuring the pelvic splanchnic nerves, can lead to urinary retention or fecal incontinence.

Beyond RHS (CN VII), the mechanism of direct viral invasion of motor nuclei can also cause other cranial nerve palsies, such as ophthalmoplegia (CN III, IV, VI) (Figure S2). This demonstrates that segmental motor palsy is a multifactorial consequence of viral spread, inflammation, and neuronal injury.

## 5 Diagnosis

The diagnosis of classic HZ can be made on clinical grounds alone. In contrast, the diagnosis of atypical cases requires confirmation through various laboratory testing methods. With technological advancements, diagnostic approaches for HZ are evolving toward greater precision, rapidity, and convenience (Figure S3).

### 5.1 Past Diagnostic Methods

The diagnosis of HZ is primarily based on its classic clinical presentation: unilateral, dermatomal vesicles with neuropathic pain [26]. For atypical cases, laboratory testing is essential. Historically, diagnosis relied on virus isolation and the Tzanck smear. The Tzanck smear offers speed and low cost by detecting multinucleated giant cells, but a key limitation is its inability to discriminate between VZV and Herpes simplex virus (HSV) [184]. Virus isolation involves culturing vesicular fluid for 3–7 days to observe cytopathic effects [185]. However, both methods are time consuming, have low sensitivity, and are unsuitable for rapid diagnosis. The Tzanck smear lacks virus specificity, and virus isolation can yield false negatives due to poor viral viability. These limitations have driven the development of novel diagnostic approaches for HZ (Figure S3).

### 5.2 New Diagnostic Methods

The diagnostic objective at this stage is to directly detect the reactivated virus, with commonly employed methods including PCR, loop‐mediated isothermal amplification (LAMP), immunofluorescence (IF) assays, and metagenomic next‐generation sequencing (mNGS) (Figure S3). Concurrently, viral reactivation triggers a specific antibody response in the host. Commonly used serological assays for detecting these antibodies include enzyme‐linked immunosorbent assay (ELISA), fluorescent antibody to membrane antigen (FAMA), time‐resolved fluorescence immunoassay (TRFIA), and chemiluminescence immunoassay (CLIA) (Figure S3).

#### 5.2.1 PCR and LAMP

Real‐time PCR is now the gold standard for detecting VZV DNA [186, 187], offering high sensitivity and specificity using samples such as vesicular fluid [185], Cerebrospinal fluid (CSF) [188], blood [189], or saliva [143], with results typically available within 1–2 days at a cost comparable to viral culture [190]. However, it requires centralized labs and sophisticated equipment, limiting point‐of‐care use. In contrast, LAMP allows rapid isothermal nucleic acid amplification without expensive instruments [191], providing results in about 30 min and showing high agreement with real‐time PCR, making it suitable for clinical, emergency, and resource‐limited settings. Additionally, PCR‐based assays can differentiate WT VZV from the vaccine‐derived Oka strain, which is crucial for monitoring the safety of VZV (Figure S3).

#### 5.2.2 Immunofluorescence

In addition to PCR and LAMP, IF is a valuable technique for detecting VZV in cutaneous vesicular specimens. It uses specific primary antibodies that bind viral antigens, followed by fluorophore‐labeled secondary antibodies for visualization, thereby revealing antigen presence and distribution to support HZ diagnosis [192]. IF is categorized into direct and indirect formats: direct IF uses a fluorochrome‐conjugated primary antibody, while indirect IF involves an unlabeled primary antibody followed by a labeled secondary antibody [193]. For rapid VZV diagnosis, direct fluorescent antibody (DFA) staining of lesion scrapings is the preferred clinical method, offering higher sensitivity than viral culture but lower than PCR [193]. Additionally, DFA cannot differentiate between WT and vaccine‐type (vOka) VZV strains [193] (Figure S3).

#### 5.2.3 Enzyme‐Linked Immunosorbent Assay

ELISA serves as the predominant technique for detecting VZV antibodies, supported by a wide range of commercially available kits [194]. Both ELISA and IF can identify VZV‐specific IgG, IgM, and IgA [192]. While elevated IgG can be nonspecific or due to HSV cross‐reactivity, raised IgM or high IgA titers are more specific serological markers, frequently signaling VZV recurrence in the absence of cutaneous lesions [195]. A VZV–IgM titer above 1:640 typically suggests acute infection. Additionally, a Goldmann–Witmer coefficient ≥3 in aqueous humor calculated as (intraocular VZV–IgG/total intraocular IgG)/(serum VZV–IgG/total serum IgG) can also provide etiological evidence of VZV infection [195, 196]. However, conventional ELISA has limited sensitivity. While gpELISA offers improved sensitivity, it may increase false‐positive rates [196] (Figure S3).

#### 5.2.4 Fluorescent Antibody to Membrane Antigen

Owing to its extensive validation, the FAMA assay, pioneered by Williams et al., is regarded as the gold standard for VZV antibody detection [197, 198]. It identifies VZV‐specific antibodies targeting viral proteins on infected cell surfaces, which correlate with vaccine‐induced protection. Various cell lines, such as Human foreskin fibroblasts（HFF), Medical research council strain (MRC)‐5, Vero, and Raji, have been used in FAMA testing. Modified versions, including gE–FAMA and flow cytometry‐based flow FAMA, have also been developed [197]. As a result, FAMA has been established as an immune correlate of protection in early varicella vaccine studies. The FAMA assay demonstrates high predictive sensitivity, as evidenced by a study in which fewer than 2% of 131 healthy individuals with a titer ≥1:4 developed mild varicella after household exposure [197]. However, due to the need for specially trained personnel, the assay is mainly used in research (Figure S3).

#### 5.2.5 Other IgG Antibody Detection Methods

Several emerging VZV IgG detection methods include TRFIA, CLIA, LIPS, and LFIA. Currently, clinical data on these methods remain limited, and further validation is needed. TRFIA shows strong correlation with both the Merck glycoprotein ELISA and the FAMA assay [199]. Using a cut‐off of 93.3 mIU/mL against the Merck ELISA, TRFIA achieved 97.8% sensitivity and 93.5% specificity [199, 200]. Compared with FAMA (cut‐off 130 mIU/mL), it showed 90% sensitivity and 78% specificity [199, 200]. TRFIA also demonstrates higher sensitivity and specificity than most commercial kits in unvaccinated populations. CLIA combines chemiluminescence with immunoassay principles, offering a wide linear range, ease of operation, and high throughput. One study developed a CLIA using VZV gE to simultaneously detect VZV‐specific IgA, IgG, and IgM. Results showed that this method, especially when combining IgM with IgG and IgA, outperforms glycoprotein ELISA in sensitivity and specificity [201, 202]. A gE‐based LIPS system reported by Cohen et al. showed 90% sensitivity and 70% specificity compared with FAMA, indicating its potential for measuring VZV antibodies in vaccinated individuals [194, 203]. LFIA is a rapid, point‐of‐care technology using colloidal gold nanoparticles for specific antibody detection [194]. A previously reported LFIA for HSV‐2 antibodies in serum and whole blood showed sensitivity and specificity comparable to ELISA [204]. In summary, these novel technologies show promising application prospects, but large‐scale clinical studies are still needed to confirm their diagnostic performance (Figure S3).

#### 5.2.6 Metagenomic Next‐Generation Sequencing

For atypical or diagnostically challenging cases, mNGS shows significant diagnostic potential. It performs unbiased sequencing of all nucleic acids in samples like cerebrospinal fluid, directly identifying VZV without needing prior hypothesis, which is particularly valuable for critically ill patients with complex infections [205, 206]. Limitations include relatively high cost, longer turnaround time than targeted PCR, and the need for advanced bioinformatics. Despite these, mNGS is a transformative tool for challenging, rare, or mixed infections where conventional methods may fail (Figure S3).

## 6 Therapeutic Advances

HZ is a multifactorial disease in which active viral replication, host immune responses, and subsequent neural injury jointly determine clinical outcomes. The preceding sections highlight that many severe manifestations and complications arise when viral replication is insufficiently controlled or when inflammation becomes dysregulated. These observations indicate that effective management must be grounded in interventions targeting the underlying disease mechanisms rather than symptom relief alone. On this basis, the following section focuses on recent therapeutic advances, beginning with antiviral strategies that directly inhibit VZV replication and extending to approaches for complication management and prevention.

### 6.1 Antiviral Drugs: Targeting the Viral Replication Stage

**FIGURE 5:**
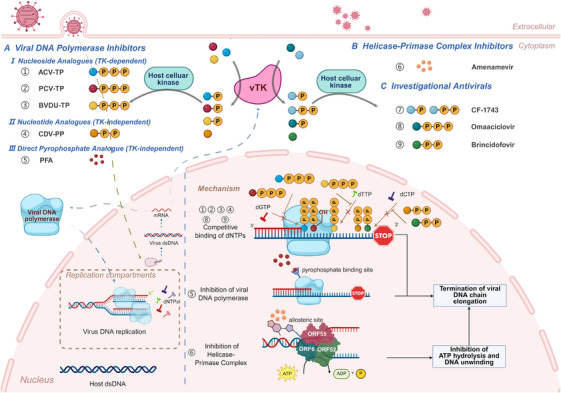
Antiviral drug classes used in the treatment of HZ. This figure summarizes the major classes of antiviral agents used for the treatment of HZ, including DNA polymerase inhibitors, helicase–primase complex inhibitors, and emerging antiviral strategies. The diagram highlights key molecular targets involved in inhibiting VZV replication. *Abbreviation*: HZ, herpes zoster.

As delineated in the above section, the active replication of VZV is the direct cause of primary varicella and the acute phase of HZ. The cornerstone of managing these active infections lies in antiviral drugs that specifically target and inhibit the viral replication machinery. This section will systematically review these agents, categorizing them based on their molecular targets within the viral DNA synthesis pathway, which primarily includes the viral DNA polymerase and the helicase–primase complex (Figure 5).

#### 6.1.1 Viral DNA Polymerase Inhibitors I: Nucleoside Analogues

The most widely used anti‐VZV drugs are nucleoside analogues, which ultimately target the viral DNA polymerase after being activated by phosphorylation.

##### 6.1.1.1 ACV and VACV

The first‐line antiviral agent for managing VZV infections is ACV [13, 207]. Furthermore, it exhibits potent and selective inhibition against herpes simplex virus type 1 [208] (HSV‐1), type 2 [208] (HSV‐2), and Epstein–Barr virus [209], while demonstrating only weak inhibitory activity against human cytomegalovirus [210] (HCMV). Currently, ACV and its L‐valyl ester prodrug, VACV, remain the gold‐standard therapeutics for the management and prevention of diseases associated with VZV. The therapeutic mechanism of ACV against VZV infection can be delineated by a two‐step process: “virus‐targeted activation” followed by “irreversible DNA chain termination.” The initial step involves selective intracellular activation within VZV‐infected cells. ACV is first monophosphorylated to ACV‐MP by the VZV‐encoded TK, encoded by the ORF36 gene [211]. This virus‐specific phosphorylation is the critical rate‐limiting step that confines the drug's subsequent activity to infected cells. The monophosphate metabolite is then sequentially converted to the diphosphate form (ACV‐DP) by host cellular guanylate kinase [211], and finally to the active triphosphate form (ACV‐TP) by a suite of cellular kinases [211] (Figure 5). In the subsequent step, ACV‐TP, a structural analog of deoxyguanosine triphosphate (dGTP), functions as a competitive substrate for the viral DNA polymerase (Figure 5). It is incorporated into the growing DNA chain in place of the natural nucleotide dGTP [211]. The incorporated ACV molecule lacks a 3'‐hydroxyl group, which precludes phosphodiester bond formation with the next incoming nucleotide and thus arrests DNA chain elongation [211, 212] (Figure 5). Furthermore, the incorporated ACV‐TP residue cannot be excised by the proofreading 3′→5′ exonuclease activity associated with the viral DNA polymerase, resulting in the irreversible termination of viral DNA synthesis (Figure 5). Regarding pharmacokinetics, ACV is characterized by low oral bioavailability (15–30%) and poor aqueous solubility, necessitating high‐dose, frequent administration to maintain therapeutic plasma concentrations [213, 214]. For immunocompromised patients, intravenous ACV remains the standard treatment, as it enables rapid attainment of higher plasma drug concentrations [215]. Meanwhile, to overcome ACV's limitations, VACV was developed by introducing an L‐valyl ester moiety [216, 217]. This modification allows it to utilize the hPEPT1 transporter for efficient intestinal uptake, thereby achieving a high oral bioavailability of 54% [217]. Following absorption into the systemic circulation, VACV is rapidly hydrolyzed by intestinal esterases to yield ACV and L‐valine. This prodrug strategy retains the original antiviral profile of ACV while substantially simplifying the dosing regimen. Substantial clinical evidence supports the comparable safety of VACV to ACV in the treatment of patients with HZ. Its superior adherence profile, attributable to reduced dosing frequency, solidifies VACV as a first‐line therapeutic option for VZV infections [217]. When clinical failure is suspected (e.g., no improvement or continued progression of disease after 10–21 days of adequate ACV therapy), the emergence of a resistant viral strain should be strongly suspected. In such scenarios, genotypic resistance testing involving PCR amplification and subsequent sequencing of the TK and DNA pol gene represents the preferred diagnostic approach [189].

##### 6.1.1.2 Penciclovir and FAM

**FIGURE 6:**
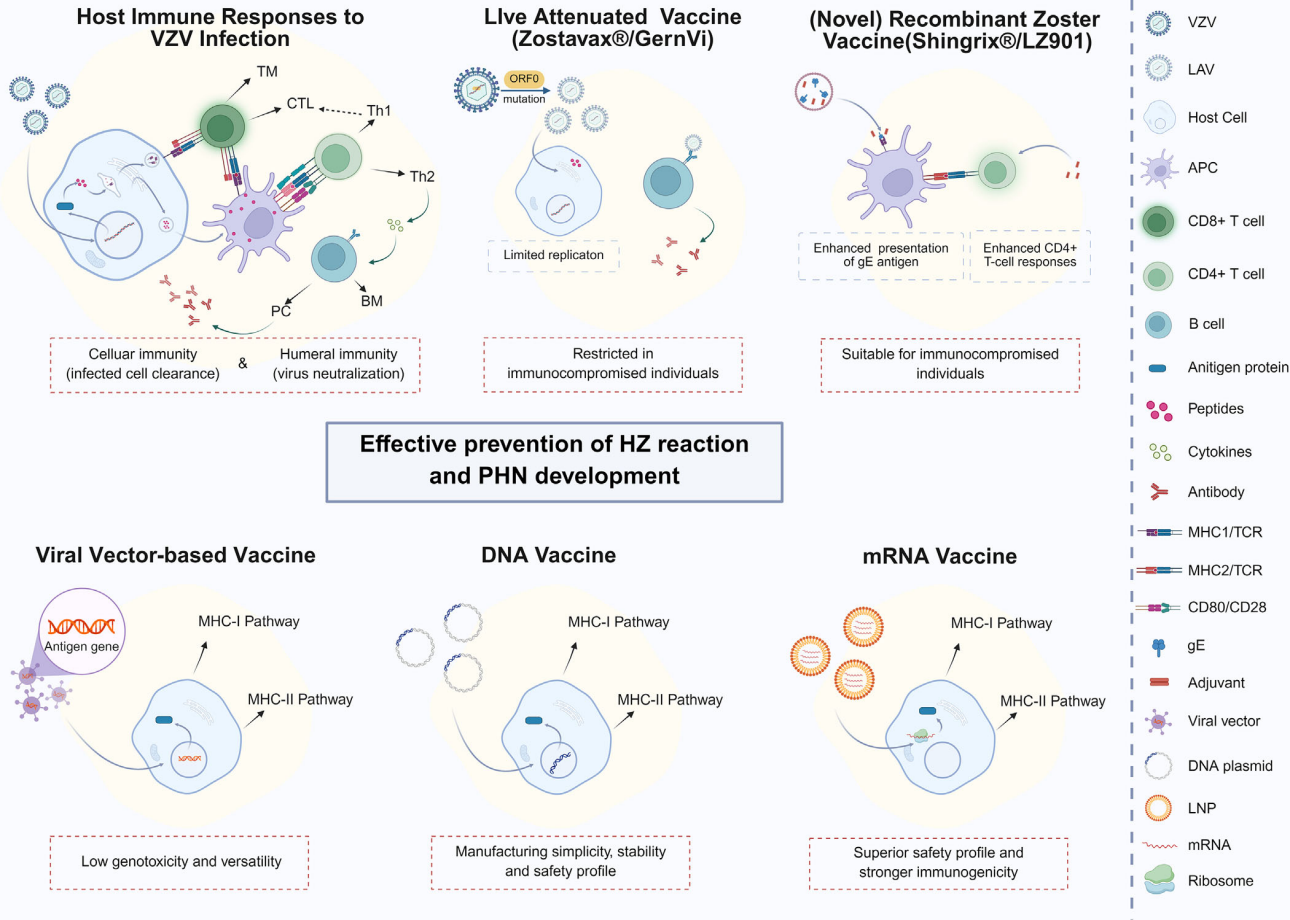
Effective prevention of HZ reaction. The main vaccines currently used for the treatment of HZ include the ZVL and the RZV that have been marketed, as well as the novel recombinant subunit vaccine, Ad‐vector vaccine, DNA vaccine, and mRNA vaccine that are under development. *Abbreviations*: ZVL, zoster vaccine live; RZV, recombinant zoster vaccine; Ad‐vector, adenovirus‐vector; DNA, deoxyribonucleic acid; mRNA, messenger RNA.

Penciclovir (PCV) is an acyclic nucleoside analog structurally related to ACV and ganciclovir, characterized by a methylene group replacing the ether oxygen in its side chain [217] (Figure 6). It shares a similar antiviral mechanism with ACV against VZV. Both prodrugs rely on initial phosphorylation by the viral TK within infected cells, a crucial step confining their activation to the site of infection (Figure 5). Subsequent phosphorylation by cellular kinases yields the active triphosphate forms, ACV‐TP and PCV triphosphate (PCV‐TP), which competitively inhibit viral DNA polymerase by acting as alternatives to the natural substrate dGTP [24, 95] (Figure 5). Despite this overarching similarity, key distinctions define their modes of action. First, PCV exhibits a greater binding avidity for HSV TK than ACV [20], leading to significantly higher intracellular levels of PCV‐TP [217, 218]. Second, PCV‐TP demonstrates greater stability in HSV‐infected cells, resulting in an intracellular half‐life 10‐ to 20‐fold longer than that of ACV‐TP [217, 218]. A critical mechanistic difference lies in their chain‐terminating behavior. The presence of two hydroxyl groups on the acyclic chain of PCV‐TP distinguishes it from ACV‐TP, which lacks the 3′‐hydroxyl group necessary for chain elongation and thus acts as an obligate terminator. This structural feature allows for limited DNA strand elongation after incorporation, classifying PCV‐TP as a short‐chain terminator rather than an absolute blocker of synthesis [217, 218]. Nevertheless, under conditions mimicking HSV‐infected cells, PCV‐TP proves more effective than ACV‐TP in DNA chain elongation assays, a potency attributed to its higher intracellular concentration and prolonged half‐life, which collectively offset the fact that HSV DNA polymerase has a higher inherent affinity for ACV‐TP [217, 219]. To enable systemic therapy, FAM, the diacetyl ester prodrug of 6‐deoxypenciclovir, was developed [220]. After oral intake, FAM undergoes rapid deacetylation in the intestinal wall by esterases, followed by oxidation at the 6‐position catalyzed by hepatic aldehyde oxidase, which converts it into the active metabolite, PCV [216, 220]. This efficient metabolic conversion confers a high oral bioavailability of approximately 77%. FAM demonstrates a favorable safety profile and exhibits broad‐spectrum efficacy against HSV‐1, HSV‐2, and VZV. Its approved use encompasses the management of HZ in both immunocompetent adults and immunocompromised patients [216, 220, 221]. However, similar to valaciclovir, FAM is not currently approved for pediatric use and may, in rare instances, be associated with adverse effects such as headache, confusion, and nausea.

##### 6.1.1.3 Brivudine

Brivudine (BVDU), a cyclic nucleoside analogue, is employed in clinical practice in several European countries for the management of HZ. Its antiviral activity profile demonstrates excellent efficacy against VZV [222] (Figure 5). This high selectivity is attributed to the specific phosphorylation of BVDU by the VZV‐encoded TK to its monophosphate (BVDU‐MP) and diphosphate (BVDU‐DP) forms [222, 223] (Figure 5). In contrast, HSV‐2 TK phosphorylates BVDU only to the monophosphate level, requiring subsequent phosphorylation by cellular TK. The active metabolite, BVDU triphosphate (BVDU‐TP), is generated via catalysis by nucleoside diphosphate kinase. BVDU‐TP competes with deoxythymidine triphosphate for binding to viral DNA polymerase, acting as an alternative substrate that is subsequently incorporated into viral DNA. This results in the loss of DNA structural and functional integrity, thereby effectively inhibiting viral DNA synthesis. Orally administered BVDU exhibits approximately 40% bioavailability, with about 70% of the absorbed dose undergoing rapid first‐pass hepatic metabolism to bromovinyluracil. In immunocompetent adults, BVDU demonstrates significant clinical efficacy by inhibiting the formation of new lesions and preventing PHN. Its once‐daily dosing regimen offers convenience comparable to other anti‐VZV agents, including ACV, VACV, and FAM. A recent retrospective study on analgesic efficacy reported significant pain reduction by Day 3 with BVDU, by Day 7 with FAM, and within 2–3 weeks with VACV, with no severe adverse events reported across the treatment groups. These findings, particularly its early pain control and simplified dosing, support the use of BVDU as a preferred option for severe HZ [222, 223]. Although generally well‐tolerated, BVDU may cause adverse effects such as gastrointestinal disturbances, renal dysfunction, elevated liver enzymes, and reversible hematological changes. Importantly, coadministration with 5‐fluorouracil or other 5‐fluoropyrimidines is contraindicated due to the potential for life‐threatening drug interactions.

#### 6.1.2 Viral DNA Polymerase Inhibitors II: Direct Pyrophosphate Analogue

Foscarnet sodium (PFA, Foscavir), a pyrophosphate analogue, directly targets the viral DNA polymerase by occupying its pyrophosphate binding site, thus preventing further DNA chain elongation [224, 225] (Figure 5). For ACV‐resistant (ACV‐R) VZV infections resulting from viral TK mutations, foscarnet is a first‐line therapy due to its unique mechanism, which does not require activation by viral thymidine kinase [35] (Figure 5). Intravenous foscarnet is recommended as an alternative antiviral regimen for immunocompromised patients with suspected or confirmed ACV‐R VZV infections, particularly in severe or disseminated cases. Dosage adjustment is required for individuals with renal dysfunction [225]. Important adverse effects include nephrotoxicity and ulcerative toxicity of the urogenital mucosa, necessitating rigorous monitoring during treatment [225].

#### 6.1.3 Viral DNA Polymerase Inhibitors III: Nucleotide Analogues

The United States Food and Drug Administration (US FDA) approved the cyclic nucleotide phosphonate cidofovir (CDV) in 1996 for IV treatment of AIDS‐related cytomegalovirus (HCMV) retinitis [226, 227] (Figure 5). The agent's broad‐spectrum anti‐DNA virus activity leads to its frequent off‐label use, with IV or topical administration for serious infections caused by pathogens like HSV, polyomaviruses, adenoviruses (Ads), poxviruses, and Human papillomaviru (HPV). For VZV strains resistant to first‐line agents, CDV serves as a crucial salvage therapy. The drug's distinct mechanism stems from its phosphonate moiety, which obviates initial viral TK phosphorylation. Following activation by cellular enzymes to its diphosphate form (CDVpp), it functions as both a competitive inhibitor and an alternative substrate for viral DNA polymerase (Figure 5). This incorporation causes delayed chain elongation and premature termination, a process favored by CDVpp's pronounced selectivity for the viral over the host polymerase [226, 227]. A key pharmacokinetic advantage stems from its metabolic conversion into CDVp‐choline adducts, which form a long‐lasting intracellular depot. This reservoir enables sustained release of CDVp and CDVpp, prolonging the intracellular half‐life and permitting once‐weekly dosing to maintain antiviral activity.

#### 6.1.4 Inhibitors of the Helicase–Primase Complex

Helicase–primase inhibitors (HPIs) were approved in Japan in September 2017 for the treatment of HZ [228, 229], although a Phase I clinical trial of this drug class in the United States was halted due to safety concerns [229, 230]. Composed of helicase, primase, and a cofactor protein in a 1:1:1 ratio, the herpesvirus‐encoded complex exhibits DNA unwinding, ssDNA‐stimulated ATPase, and primase activities, which collectively establish its role as an indispensable engine for viral DNA replication (Figure 5). In VZV, the corresponding genes are ORF55 (helicase), ORF6 (primase), and ORF52 (cofactor), which share high homology with UL5/UL52/UL8 in HSV‐1 and UL105/UL70/UL102 in HCMV. Amenamevir, an HPI, inhibits the coupling of Adenosine triphosphate (ATP) hydrolysis to DNA unwinding by binding to an allosteric site on the complex via its oxadiazole phenyl backbone, thereby suppressing viral DNA elongation [231]. As this mechanism is independent of viral TK, it remains active against TK‐deficient mutants and exhibits synergistic effects in vitro when combined with nucleoside analogues, suggesting its potential for combination therapy in severe or immunocompromised cases. Amenamevir demonstrates nanomolar‐level activity against both VZV and HSV, exhibits low cellular toxicity, and shows good oral bioavailability alongside significant viral load reduction in various murine HSV models [231]. To confirm target specificity, researchers serially passaged VZV under increasing drug concentrations and identified resistant mutants harboring amino acid substitutions in ORF55 (N336K, R446H) and ORF6 (N939D) [232]. These mutants exhibited impaired replicative fitness, confirming that the anti‐VZV effect of amenamevir is specifically mediated through inhibition of the helicase–primase complex [232]. Currently, global Phase III trial data are still lacking, and evidence regarding its use in immunocompromised populations, pediatric patients, and central nervous system infections remains limited, underscoring the need for international multicenter studies to evaluate its safety in broader populations [232]. Pritelivir, another HPI, has also demonstrated potent anti‐VZV activity in clinical trials, circumventing resistance to existing antivirals and showing particular therapeutic potential for refractory infections in immunocompromised patients [229].

#### 6.1.5 Investigational Antivirals With Novel Mechanisms

##### 6.1.5.1 Farniclovir Hydrochloride and its Parent Compound CF‐1743

Farniclovir hydrochloride (FV‐100) is an oral prodrug of the bicyclic nucleoside analogue CF‐1743 [233, 234]. The mechanism of action of CF‐1743 is highly unique and strictly dependent on VZV. It is phosphorylated by the VZV‐encoded thymidine kinase, which unusually catalyzes its conversion in a single step to both the monophosphate and diphosphate forms [234]. However, unlike conventional nucleoside analogues such as BVDU, current studies have not detected the triphosphate form of CF‐1743 in infected cells, and the ultimate molecular target of its active form is not the viral DNA polymerase—the precise mechanism remains under active investigation. This distinctive pathway confers exceptionally high selectivity against VZV, with minimal inhibition observed against other herpesviruses. CF‐1743 exhibits outstanding potency and selectivity, inhibiting VZV at sub‐nanomolar EC_50_ values, significantly surpassing the activity of ACV and BVDU [235] (Figure 5).

##### 6.1.5.2 Valomaciclovir Stearate

Valomaciclovir stearate, a dual prodrug of omaciclovir, shares a mechanism of action with ACV, as both depend on viral TK for their initial phosphorylation and activation [236, 237]. The active metabolite competes with natural nucleotides for viral DNA polymerase but differs from ACV in that it does not function as an obligate chain terminator. The presence of a potential 3′‐hydroxyl group on its side chain permits limited DNA chain elongation, a distinctive feature that may contribute to a differing resistance profile compared with ACV [237]. The intracellular half‐life of valomaciclovir is substantially longer than that of ACV, supporting a once‐daily dosing regimen [237].

##### 6.1.5.3 Modified Nucleotide Analog Precursor

Brincidofovir is a hexadecyloxypropyl ester prodrug of the nucleotide analogue CDV. It is intrinsically inactive and must undergo intracellular cleavage of its lipid moiety to release CDV monophosphate, which is subsequently phosphorylated by cellular kinases to its active form, CDV diphosphate [238]. This active metabolite functions as a dCTP analogue, competitively inhibiting viral DNA polymerase and being incorporated into the DNA chain, ultimately leading to delayed and premature termination of viral DNA synthesis (Figure 5). Notably, although brincidofovir encountered setbacks in Phase III clinical trials for cytomegalovirus prophylaxis due to gastrointestinal adverse events and efficacy concerns, case reports have successfully demonstrated its effectiveness in treating disseminated ACV‐ and CDV‐resistant VZV infection in HCT recipients [238, 239]. Following its acquisition by SymBio Pharmaceuticals, the global rights to brincidofovir may potentially reposition it as a therapeutic option in the management of resistant HSV infections.

### 6.2 Treatment for Complications: Classified by Pathological Mechanisms

#### 6.2.1 Complications Driven by Continuous Viral Replication

Complications driven by persistent VZV replication, including encephalitis/meningitis, DHZ, vasculitis, and acute retinal necrosis (ARN), necessitate a foundational strategy of immediate, high‐dose intravenous ACV to rapidly halt viral replication and prevent irreversible damage [240, 241, 242, 243]. For VZV encephalitis/meningitis, prompt IV ACV (10 mg/kg every 8 h) is essential, often with a 14–21 day course, alongside management of cerebral edema and seizures [240, 241]. DHZ and VZV vasculitis are similarly treated with IV ACV [243, 244, 245]; vasculitis typically requires adjunctive corticosteroids to control inflammatory vascular damage [177]. The management of ARN is particularly aggressive, requiring a multipronged approach with initial IV ACV transitioning to prolonged oral therapy for at least 3 months to prevent bilateral involvement [246, 247]. This systemic treatment must be augmented by intravitreal antiviral injections to achieve therapeutic intraocular levels [247, 248], with urgent surgical intervention (e.g., vitrectomy) if retinal detachment occurs or is threatened [246, 247]. Across all these severe manifestations, initiating antiviral therapy empirically upon clinical suspicion is critical and should not be delayed for confirmatory testing [242].

#### 6.2.2 Complications Driven by Inflammatory Responses

The treatment for complications driven primarily by inflammatory responses to VZV, such as HZO with deep keratitis/uveitis and severe neuritis like RHS, centers on a dual strategy of combined antiviral and corticosteroid therapy to suppress both the virus and the damaging immune response [16, 249, 250, 251]. For HZO complicated by deep keratitis or uveitis, management includes oral VACV or FAM at standard doses [16], augmented by topical corticosteroid eyedrops (e.g., prednisolone acetate) to control intraocular inflammation [249]. The initiation of topical steroids, however, requires caution; it should only be started once the corneal epithelium is intact or under adequate antiviral coverage to prevent the exacerbation of epithelial keratitis or viral reactivation, and once inflammation is controlled, a very gradual taper is mandatory to avoid rebound [249]. In the case of RHS, first‐line treatment consists of high‐dose oral VACV (1 g three times daily) or FAM (500 mg three times daily), with intravenous ACV reserved for severe cases or when oral administration is not feasible [251]. Crucially, oral prednisone is a critical component, as multiple randomized controlled trials and meta‐analyses have consistently demonstrated that combination therapy with antivirals and corticosteroids significantly improves the rate of complete facial functional recovery and reduces the risk of chronic pain compared with antiviral monotherapy [250, 251]. This combined immunomodulatory and antiviral approach is fundamental to managing these inflammation‐driven conditions effectively.

#### 6.2.3 Complications Resulting From Sequelae of Nerve Damage

The management of complications resulting from sequelae of nerve damage, such as PHN and motor nerve palsy [252], focuses on alleviating chronic symptoms after active viral replication has ceased, with first‐line PHN treatments including calcium channel α2‐δ ligands (gabapentin, pregabalin), tricyclic antidepressants (amitriptyline), and topical 5% lidocaine patches to modulate central and peripheral pain signaling [253, 254]. For refractory cases, second‐line options encompass the high‐concentration 8% capsaicin patch for prolonged peripheral pain relief and local injections of botulinum toxin type A to inhibit neurotransmitter release [253, 254]. Beyond pharmacotherapy, interventional procedures such as nerve blocks or epidural steroid injections can be utilized, while severe, intractable pain may require neuromodulation techniques like spinal cord or peripheral nerve stimulation [253, 254]. Notably, continuous epidural block with local anesthetics and corticosteroids during AHZ has been associated with a lower incidence of PHN at 3 months compared with other strategies [255, 256]. For motor nerve palsy, the therapeutic goal is to maximize functional recovery through nutritional support with B vitamins like methylcobalamin, physical therapy, and rehabilitation, with reconstructive surgery considered for severe, persistent cases [257]. Several systemic and topical agents, as well as procedural interventions, remain under investigation for future PHN management [255, 256].

## 7 Preventive Strategies

HZ vaccine serves as a critical preventive intervention targeting the reactivation phase. It functions by augmenting VZV‐specific cellular immunity, thereby enabling the immune system to more effectively surveil and suppress the reactivation of latent virus. Approved HZ vaccines globally fall into two classes: ZVL and RZV. Furthermore, numerous vaccine candidates are currently under development or in clinical trials, including novel recombinant subunit vaccines, Ad vector‐based vaccines, DNA vaccines, and mRNA vaccines, among others (Figure 6).

### 7.1 Live‐Attenuated Zoster Vaccine

ZVL represents the first and currently the only herpesvirus vaccine available. This class of vaccines utilizes attenuated VZV strains, such as the Oka strain, to induce immune responses in the host [258] (Figure 6). The attenuation of the live‐attenuated vOka vaccine is primarily mediated by its impaired replication in the skin [259], resulting from cumulative mutations in key viral genes—most critically in ORF62, which encodes the major transactivator IE62, leading to disrupted interaction with host epidermal pathways such as failure to upregulate keratin K15, along with contributory roles from mutations in ORF0, ORF31(gB), and reduced expression of ORF14 (gC), collectively diminishing cell‐free virion production, transmission, and establishment of latency while preserving immunogenicity [260, 261, 262]. The molecular mechanism of ZVL involves the administration of a viable but pathogenically weakened virus, which undergoes limited replication in the host [263]. This process mimics natural infection and elicits durable humoral and cellular immune responses. The immune response provoked by these vaccines comprises the activation of CD4+ and CD8+ T cells alongside the production of neutralizing antibodies [264, 265]. In the case of VZV live attenuated vaccines, the immune system recognizes viral antigens and establishes immunological memory against VZV. This enables a rapid and effective response upon subsequent exposure to WT virus, thereby preventing disease occurrence [266] (Figure 6).

Zostavax, the first live attenuated HZ vaccine, was initially licensed in the United States in 2006 and has been incorporated into population‐based immunization programs in several high‐income countries, including Australia, the United Kingdom, and the United States. As a ZVL based on the Oka/Merck strain, it contains a substantially higher viral titer compared with the varicella vaccine. It is designed to reactivate and boost the declining VZV‐specific cellular immunity in older adults [267]. The efficacy of Zostavax was shown to be highly dependent on the recipient's age. Efficacy against HZ was approximately 69% in subjects aged 50–59 years, but was progressively lower in older groups—64% for ages 60–69 years and only 18% for those 80 years or older. In adults over 60 years of age, it was about 51% effective in preventing PHN [268, 269, 270]. This decline in efficacy is attributed to the reduced responsiveness of the aging immune system to live vaccines. In addition to Zostavax, several other ZVLs, including those developed by SK Bioscience (South Korea), Baike Biotech's GernVi (China), and the Oka/Biken strain (Japan), have been approved and are available in various countries. However, ZVL products still possess inherent limitations. For instance, Zostavax is contraindicated in immunocompromised or immunodeficient individuals, such as organ transplant recipients, patients undergoing chemotherapy, or those with HIV/AIDS, due to the theoretical risk of disseminated vaccine‐related infection. Overall, while ZVL have demonstrated substantial efficacy in immunocompetent older adults, their utility remains limited in immunocompromised populations due to safety concerns regarding potential reversion to virulence (Figure 6).

### 7.2 Recombinant Zoster Vaccine

Shingrix represents a major breakthrough in HZ prevention and is currently the only widely available RZV [271] (Figure 6). This nonlive subunit vaccine, which first received approval in Canada and the United States in 2017, comprises a key viral antigen: the gE, the most prevalent glycoprotein on the VZV envelope that is critical for replication and cell‐to‐cell spread. The antigen is formulated with the AS01B adjuvant system to enhance the immune response [271]. As the predominant VZV antigen targeted by the immune response, gE serves as an ideal target for inducing protective immunity. The molecular mechanism of RZVs such as Shingrix involves the administration of the recombinant gE antigen together with the AS01B adjuvant system. This adjuvant enhances antigen uptake and presentation by antigen‐presenting cells, leading to potent activation of both T‐cell and B‐cell immune responses. Specifically, AS01B promotes a robust and durable CD4+ T‐cell‐mediated immune response, which is critical for protecting older adults against HZ [76]. Unlike live attenuated vaccines, RZV contains no live viral components, thereby eliminating any risk of viral replication or reversion to virulence and making it suitable for use in immunocompromised individuals. The vaccination schedule consists of two intramuscular doses administered 2–6 months apart. A clinical study (NCT02581410) demonstrated that Shingrix elicits a strong immune response even in older adults previously vaccinated with Zostavax, supporting its use as an effective sequential booster strategy [272]. The pivotal Zoster efficacy study (ZOE)‐50 and ZOE‐70 trials demonstrated consistently high efficacy for the vaccine across all age groups. Overall protection exceeded 90% in adults aged 50 years and above, with rates of 97.2, 91.3, and 80.3% in the 50–59, 70–79, and ≥80‐year‐old cohorts, respectively [273, 274]. This high level of protection remained consistent across sex, geographic region, and racial groups. Protection is also sustained, with vaccine efficacy against HZ remaining at 81.6% between 5.6 and 9.6 years postvaccination. Shingrix also demonstrates high efficacy in preventing severe HZ‐related complications, conferring 88.8–91.2% protection against PHN and significantly reducing the risks of HZ‐associated vascular events, disseminated disease, and ocular or neurological complications [275]. Importantly, research indicates that HZ infection substantially increases the short‐term risk of stroke, and vaccination with Shingrix can mitigate this risk, adding substantial public health value. As a nonlive vaccine, Shingrix is considered safe for immunocompromised individuals and remains equally effective in those with common chronic conditions such as diabetes, chronic kidney disease, and cardiopulmonary diseases who are at elevated risk of HZ [276, 277]. For example, patients with asthma and chronic obstructive pulmonary disease face a 24 and 41% increased risk of HZ, respectively, making them priority groups for vaccination [33].

### 7.3 Novel Recombinant Subunit Vaccines

Novel recombinant subunit vaccines are designed to elicit broader and more durable protective immune responses through precisely engineered antigen structures optimized for immunogenicity, in combination with potent adjuvant systems (Figure 6). The objective of such vaccines is to overcome the limitations of conventional subunit vaccines in inducing robust cellular immune responses, while retaining their inherent favorable safety profile. This approach is particularly important for vulnerable populations, including elderly individuals with age‐related immune decline and immunocompromised patients. The key challenges in developing recombinant subunit vaccines lie in the selection of candidate antigens such as gE, gB, and gH and the optimization of adjuvant systems. For example, Jin et al. developed a gE–Fc fusion protein vaccine, LZ901, and demonstrated through clinical trials that in adults aged 50 years and older, LZ901 was noninferior to the licensed recombinant subunit vaccine HZ/su in inducing CD4+ and CD8+ T‐cell responses [278]. Notably, it elicited significantly higher response rates in both CD4+ and CD8+ T cells [278, 279]. Wang et al. also identified that the BK‐02 adjuvant system, which combines the TLR9 agonist BK‐02C (CpG2006) with a squalene‐based oil‐in‐water emulsion (BK‐02 M/MF59), triggered potent gE‐specific CD4+ and CD8+ T‐cell responses along with a mixed Th1‐polarized profile in murine models [280].

### 7.4 Viral Vector‐Based Vaccines

Ad vector‐based vaccines deliver antigen‐encoding genes into host cells, thereby inducing an immune response [281, 282] (Figure 6). Upon entering host cells, viral vector‐based vaccines enable the transcription and translation of encoded VZV antigen genes into viral proteins [283]. These endogenously expressed antigens are subsequently presented to the immune system, primarily through the MHC‐I pathway, thereby eliciting a cytotoxic T lymphocyte (CD8+ T cell) response. Concurrently, they can also activate helper T cell (CD4+ T cell) and humoral immune responses [283]. Compared with other viral vectors, the Ad vector offers distinct advantages, including low genotoxicity, a favorable safety profile, and the versatility to be engineered either as replication‐deficient vectors or selectively replicating oncolytic viral vectors. A candidate HZ vaccine based on a chimpanzee Ad (ChAd) vector encoding VZV gE has been developed. Studies demonstrated that it elicits a stronger gE‐specific T‐cell response in mice compared with the established vaccines Shingrix and Zostavax [284].

### 7.5 DNA Vaccine

DNA vaccines elicit immune responses by delivering plasmid vectors encoding target antigen genes into the host [285, 286] (Figure 6). Following intramuscular or intradermal administration, plasmid DNA is taken up by host cells and transported into the nucleus, where it undergoes transcription to generate mRNA. The synthesized mRNA is transported to the cytoplasm, where it serves as a template for the production of VZV antigenic proteins. A subset of these internally generated antigens undergoes proteolytic processing and is presented on the MHC‐I pathway, triggering the activation of CD8+ T lymphocytes specific to the antigens and initiating cellular immunity. In parallel, certain antigens can be internalized by professional antigen‐presenting cells and presented via the MHC Class II pathway, leading to the stimulation of CD4+ T helper cells and enhancement of humoral immune responses [286]. These platforms are associated with a number of benefits, such as a well‐tolerated nature, rapid development timelines, and a proven capacity for eliciting potent cellular immunity [287]. However, their immunogenicity is relatively limited when administered alone, particularly in large animal models and humans [288]. To address this limitation, various delivery strategies have been developed to enhance DNA uptake, expression, and immunogenicity, such as gene guns, needle‐free injection devices, and in vivo electroporation. For example, Kim et al. immunized C57BL/6 mice with recombinant DNA plasmids containing VZV gE, IE63, or IE62 genes. All three DNA vaccines induced strong VZV‐specific CD4+ T‐cell responses [289].

### 7.6 mRNA Vaccine

mRNA vaccines are developed through in vitro transcription of mRNA encoding target foreign genes, which is then delivered into host cells via specific delivery systems [290, 291] (Figure 6). This process enables the synthesis of the target proteins, thereby triggering antigen‐specific immune responses. Compared with DNA vaccines, mRNA vaccines exhibit a superior safety profile and enhanced immunogenicity, positioning them as a prominent focus in vaccine research. Following delivery, the lipid nanoparticle (LNP)‐encapsulated mRNA is internalized by host cells, including antigen‐presenting cells and myocytes. Within endosomes, the LNP releases mRNA into the cytoplasm, where cellular ribosomes translate it into antigenic proteins such as those derived from VZV [292, 293]. These endogenously produced antigens are processed and presented on the cell surface via both MHC Class I and Class II pathways, thereby activating CD8+ and CD4+ T cells, respectively, and stimulating B cells to produce high levels of neutralizing antibodies [292, 293]. In 2020, Monslow et al. designed an mRNA vaccine encoding a C‐terminally truncated form of VZV gE to modulate its intracellular sorting and expression. In rhesus macaques, immunization with either a single dose of 100–200 µg or two doses of 50 µg of the gE mRNA vaccine elicited immune responses comparable to those induced by two doses of a 50 µg recombinant gE protein subunit vaccine adjuvanted with AS01B, and significantly stronger than those induced by ZVL [294]. In another study, a novel VZV mRNA vaccine candidate, ZOSAL, was designed, which utilizes full‐length gE to ensure broad T‐cell epitope coverage across the entire protein [295]. In both rhesus macaques and mice, ZOSAL elicited stronger VZV‐specific Th1‐type T‐cell responses than Shingrix [295]. Vaccination with mgE@Syn‐LNP containing VZV gE mRNA also induced strong antibody and T‐cell responses in mice. The immune levels were on par with the adjuvanted recombinant gE protein and markedly higher than those observed with ZVL [296]. Overall, the application prospects of mRNA HZ vaccines are broad when various strategies are utilized to modify them [297, 298, 299].

## 8 Conclusions and Prospects

By 2050, it is projected that one in six people globally will be aged 65 years or older. Between 2019 and 2050, the number of older persons is expected to double in all regions except sub‐Saharan Africa [300]. Without widespread HZ vaccination, global projections indicate 278 million HZ cases over the next 10 years, with 10.5 million occurring in individuals aged 85 years or older [300]. Furthermore, 20.7 million persons aged 50 years and above are anticipated to develop PHN. Population‐wide estimates suggest a one‐in‐three lifetime risk of HZ for adults, while half of all individuals surviving to age 85 years will be affected by the disease [22, 301]. Therefore, effective treatment and prevention strategies for HZ are of critical importance.

**FIGURE 7:**
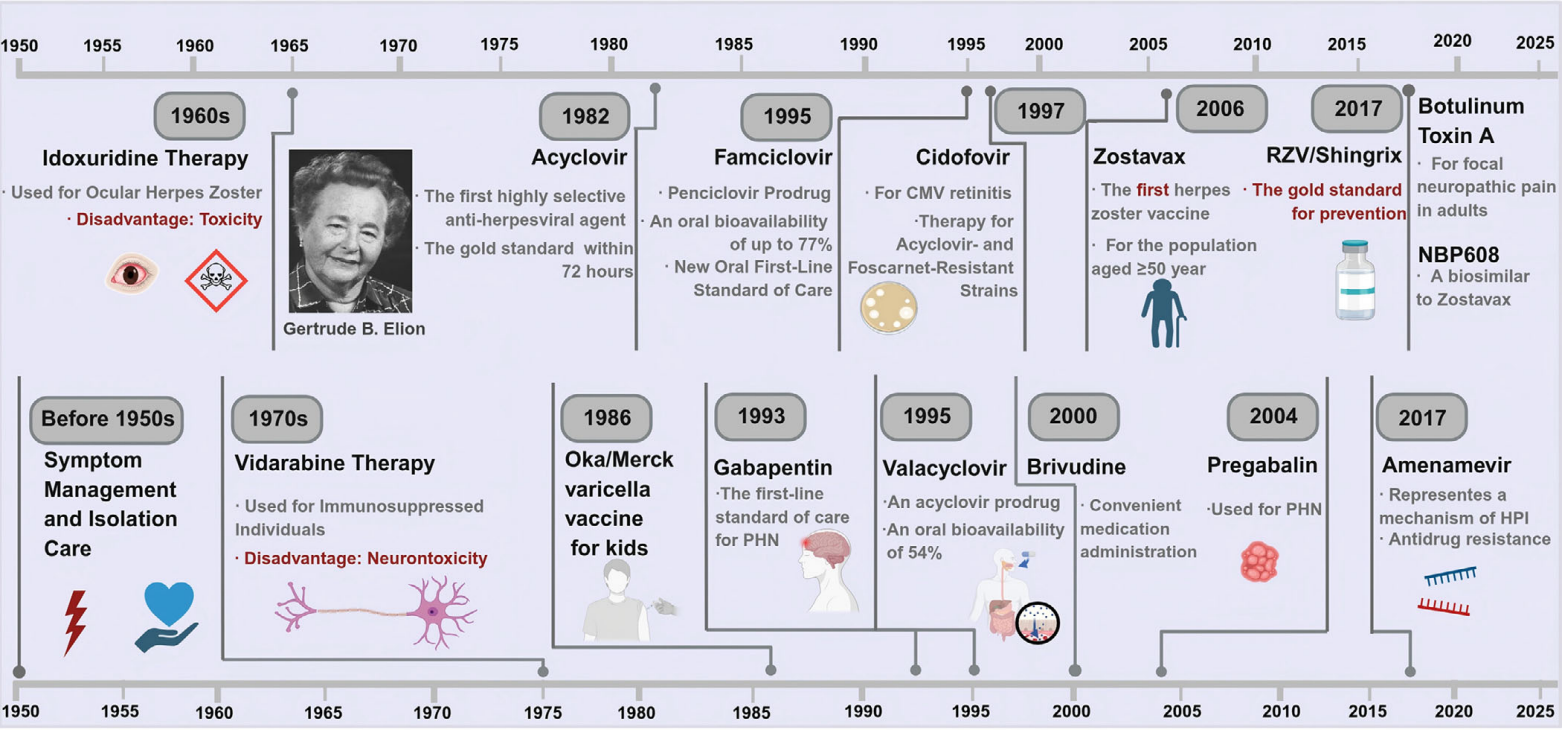
Evolution of VZV treatment and prevention strategies. Timeline depicting the transition from supportive care to targeted antivirals, pain modulators, and vaccination.

Since the initial isolation of the VZV in 1953, researchers have accumulated extensive knowledge regarding its treatment and vaccine‐based prevention [302] (Figure 7). For patients with suspected HZ, antiviral therapy should be initiated as early as possible, preferably within 72 h of rash onset, to reduce disease severity, shorten clinical duration, and alleviate pain intensity. In high‐risk patients susceptible to PHN, early adjunctive use of anticonvulsants or tricyclic antidepressants may be considered for prophylaxis [302]. Notwithstanding these interventions, current strategies for managing VZV remain suboptimal compared with advances achieved for other viral infections. An encouraging trend, however, is the expanding exploration of diverse therapeutic approaches for VZV, as reflected by the growing number of ongoing clinical trials worldwide (Table 1).

**TABLE 1:** The clinical trial landscape of antiviral therapeutics and prophylactic vaccines targeting VZV infection.

Category		Name	Country	Characteristics	Status
Antiviral drug	Nucleoside analogues	Acyclovir	United States	Inhibits viral DNA polymerase after phosphorylation by viral thymidine kinase	US FDA approval: 1985
		Valacyclovir	United States	L‐valyl ester prodrug of acyclovir that converts to acyclovir for enhanced bioavailability	US FDA approval: 1996
		Penciclovir	British	Guanosine analogue that persists in infected cells for prolonged antiviral activity after phosphorylation	US FDA approval: June 29, 1994
		Famciclovir	British	Diacetyl ester prodrug of penciclovir that hydrolyzes to penciclovir for oral administration	US FDA approval: May 31, 2012 for HZ/herpes simplex infections
		Brivudine	Germany	Thymidine analogue that selectively inhibits viral DNA replication after phosphorylation	NCT07099157 Phase IV (not recruiting)
	Direct pyrophosphate analogue	Foscavir	United States	Pyrophosphate analogue that directly inhibits viral DNA polymerase without requiring activation	NCT02151240 Phase IV (completed)
	Nucleotide analogues	Cidofovir	United States	Nucleotide analogue that directly inhibits viral DNA polymerase after phosphorylation by cellular enzymes	Preclinical status [303]
	Inhibitors of the helicase–primase complex	Amenamevir	Japan	Blocks the helicase–primase complex essential for viral DNA replication	NCT00487682 Phase III (completed)
		Pritelivir	Germany; U.S.	Prevents the unwinding and priming of viral DNA during replication	NCT02871492 Phase II (completed)
	Investigational antivirals	Farniclovir hydrochloride (FV‐100)	United States	VZV‐specific TK‐dependent activation; final target remains under investigation	NCT01327144 Phase III (completed)
		Valomaciclovir Stearate	United States	Investigational prodrug designed to be converted to penciclovir through valine esterification	NCT00831103 Phase II (completed)
Vaccine	Zoster vaccine live	Zostavax	United States	Live‐attenuated VZV (Oka/Merck); ≥19 400 PFU	US FDA approval: May 25, 2006 for adults aged ≥60 years; March 24, 2011 for adults aged 50–59 years
		SK Bioscience	South Korea	Live‐attenuated Oka/SK strain	NCT03120364 Phase III (completed); approved in 2017 in Korea for adults ≥50 years
		Baike Biotech's GernVi	China	Live‐attenuated Oka/SK strain	NCT04334577 Phase III (completed)
	Recombinant zoster vaccine	Shingrix	United States	Recombinant VZV gE, adjuvanted	US FDA: October 20, 2017 for adults aged ≥50 years; July 23, 2021 for adults ≥18 year of age who are or will be at increased risk of HZ due to immunodeficiency or immunosuppression caused by known disease or therapy
	Novel recombinant subunit vaccines	LZ901	China	A recombinant zoster vaccine consisting of a tetramer of VZV gE	NCT05750017 Phase I (recruiting)
		gE/BK‐02	China	Composed of both the TLR9 agonist BK‐02C and a squalene‐based oil‐in‐water emulsion, BK‐02M	Preclinical status [280]
		EG‐HZ	South Korea; Australia	Adjuvanted recombinant VZV gE protein	NCT04210752 Phase I (completed)
		CRV‐101	United States; South Korea	gE subunit vaccine with proprietary adjuvant	NCT03820414 Phase I (completed)
	Viral vector‐based vaccines	ChAdOx1–VZVgE	British; China	Adenoviral vaccine (ChAdOx1) encoding VZV gE	Preclinical status [284]
	DNA vaccine	VV–gE	South Korea	Recombinant DNA plasmids containing VZV gE, IE63, or IE62 genes	Preclinical status [304]
	mRNA vaccine	VZV gE mRNA/LNP	United States	An mRNA vaccine encoding a C‐terminally truncated form of VZV gE	Preclinical status [294]
		ZOSAL	China	A vaccine containing sequence‐optimized mRNAs encoding full‐length gE encapsulated in an ionizable lipid nanoparticle	Preclinical status [295]
		mgE@Syn–LNP	China	A synergistic lipid nanoparticle encapsulating mRNA shingles vaccine	Preclinical status [296]

*Data sources*: Clinical registration website (https://www.clinicaltrials.gov/).

*Abbreviations*: AIDS, acquired immunodeficiency syndrome; ChAdOx1, chimpanzee adenovirus Oxford 1; US FDA, United States Food and Drug Administration; gE, glycoprotein E; IE, immediate early protein; LNP, lipid nanoparticle; mRNA, messenger RNA; NCT, ClinicalTrials.gov identifier; PFU, plaque‐forming units; TLR9, Toll‐like receptor 9.

The development of therapeutic and preventive strategies against VZV continues to face several key challenges. (1) *A significant imbalance in viral target research*. Nearly all approved antiviral agents for VZV such as ACV, VACV, FAM, and brovudine target the viral DNA polymerase. Although brovudine also interacts with the viral TK, other potential targets, including ORF9 [138] and ORF63/70 [305], which are essential in viral latency and immune evasion, have been largely overlooked. (2) *Drug resistance*. It poses a serious clinical concern, particularly in immunocompromised patients undergoing long‐term ACV prophylaxis or treatment [216]. Resistance‐associated mutations predominantly localize to the TK and DNA polymerase genes. Once a drug‐resistant VZV infection is diagnosed, treatment options are severely limited, often restricted to intravenous agents such as foscarnet or CDV. However, the utility of these drugs is constrained by significant adverse effects, including severe nephrotoxicity and electrolyte disturbances, which lead to poor patient tolerance and complicate clinical management. Consequently, the urgent development of novel therapeutics effective against resistant VZV strains remains a critical unmet need. (3) The narrow therapeutic window and delayed diagnosis are the main bottlenecks. The golden window for antiviral therapy typically falls within 48–72 h after symptom onset. However, the early symptoms of HZ, such as pain, are often nonspecific and frequently lead to misdiagnosis. Consequently, there is an urgent need to develop rapid molecular or antigen‐based detection techniques using noninvasive samples such as saliva or skin swabs. (4) *The limitations of animal models*. Given the restricted tropism of VZV, fully replicating human HZ pathogenesis in animal models remains challenging. The rat model, including footpad and facial approaches, is widely used for studying zoster‐associated pain and PHN due to its well‐developed nervous system and stable pain responses [306]. HSV‐based mouse models can partially mimic VZV infection by inducing skin lesions and persistent pain, although pain responses are relatively weak. Pseudorabies virus models mainly study neuropathic itch rather than pain and face significant species variation limitations [306]. While the simian varicella virus infection in monkeys most closely parallels human VZV infection, ethical and cost constraints restrict its application. There is consequently a pressing need for more human‐relevant preclinical models to advance VZV research. (5) *Vaccine hesitancy*. Despite its clear advantages, the widespread adoption of RZV faces several challenges. First, even after its approval in some developing countries as early as 2019, vaccination willingness and coverage remain suboptimal [307, 308]. Second, concerns regarding vaccine safety such as rare reported cases of GBS or MOG antibody‐associated optic neuritis following RZV vaccination, albeit at very low incidence warrant continued monitoring and may partially affect vaccination confidence [309]. Furthermore, the COVID‐19 pandemic has introduced additional complexities. Some observational data suggested an increased incidence of HZ following mRNA COVID‐19 vaccination, particularly after the second dose [310]. However, a 2024 study clarified that SARS‐CoV‐2 infection itself is associated with an elevated risk of HZ, while COVID‐19 vaccination does not directly contribute to this risk [32]. This phenomenon may indicate that in older adults not vaccinated against HZ, significant immune activation events could unmask latent VZV reactivation risk underscoring the importance of prior administration of a highly effective HZ vaccine rather than attributing the risk to COVID‐19 vaccines.

Accumulating evidence on the pathogenic mechanisms of the VZV is paving the way for novel therapeutic and preventive strategies that target the critical interactions between the virus and its host. Jacobsen et al. were the first to demonstrate that VZV gC not only binds to IFN‐γ but also enhances select downstream signaling, leading to the upregulation of specific IFN‐stimulated genes [94]. This, in turn, promotes T cell adhesion and facilitates viral spread. Their findings indicate that the IFN‐γ‐binding region of gC (Y322‐S523) and its structural conformation represent potential targets for small‐molecule or antibody‐based therapeutics. Furthermore, a recent multiomics proteomic study has identified multiple host dependency factors including MPP8, ZNF280D, GRAMD1A, the PIK3CA/PIK3R1 complex, the HUSH complex, and DENND4A/C as promising targets for developing novel VZV inhibitors [311]. Additional strategies may involve developing interfacial blockers targeting virus–host protein interactions or employing agonists/gene therapies to enhance the expression or function of host restriction factors such as NPHP4 and IFI16. Research by Frances et al. [77] further established that VZV infection triggers CREB phosphorylation. The subsequent nuclear translocation of the resulting pCREB enables its interaction with the p300/CREB‐binding protein (CBP) complex, leading to apoptosis. Targeting this interaction presents a potential new antiviral strategy [77].

## Author Contributions

Lei Peng: conceptualization and original draft. Honghao Song: methodology, review, and editing. Tianying Li: data curation and formal analysis. Yuqing Ma: data curation and visualization. Chen Yan: investigation and resources. Yuhan Cao: investigation and resources. Kaiqiang Sun: supervision and project administration. Chaofeng Han: funding acquisition, review, and editing. Hongbin Yuan: conceptualization, supervision, review, and editing. All authors have read and approved the final manuscript.

## Funding

This study was supported by the National Natural Science Foundation of China (Grant No. 82302760), Shanghai Sailing Program (Grant No. 23YF1459100), Military Logistics Scientific Research Fund (AHJ21J003), and Military Clinical Key Specialty Project Fund.

## Ethics Statement

The authors have nothing to report.

## Conflicts of Interest

The authors declare no conflicts of interest.

## Supporting information




**Supplementary Figure 1**. The process of literature screening, analysis and visualization, and hotspot analysis using bibliometrics.
**Supplementary Figure 2**. Schematic diagram of complications driven by different pathogenic mechanisms.
**Supplementary Figure 3**. **Schematic overview of diagnostic approaches for HZ**. (A) Historical diagnostic pathway: when patients present with characteristic symptoms such as pathognomonic dermatomal vesicular rash, diagnosis has traditionally relied on clinical assessment; in the absence of characteristic lesions, cytological (Tzanck smear) and virological (viral isolation) assays were used. (B) Contemporary diagnostic pathway: for cases with characteristic rash, clinical diagnosis remains important but is commonly complemented or confirmed by molecular testing.

## Data Availability

The authors have nothing to report.
