# Supplementary material for: Varicella Zoster Virus Infection: Clinical Features, Molecular Pathogenesis, Treatment, and Prevention
Source: MedComm (2020). 2026 Mar 5;7(3):e70661. doi: 10.1002/mco2.70661 (PMC12963800; doi:10.1002/mco2.70661)
Supplement: Supplementary file 1 — Supplementary Figure 1. The process of literature screening, analysis and visualization, and hotspot analysis using bibliometrics. Supplementary Figure 2. Schematic diagram of complications driven by different pathogenic mechanisms. Supplementary Figure 3. Schematic overview of diagnostic approaches for HZ. (A) Historical diagnostic pathway: when patients present with characteristic symptoms such as pathognomonic dermatomal vesicular rash, diagnosis has traditionally relied on clinical assessment; in the absence of characteristic lesions, cytological (Tzanck smear) and virological (viral isolation) assays were used. (B) Contemporary diagnostic pathway: for cases with characteristic rash, clinical diagnosis remains important but is commonly complemented or confirmed by molecular testing. [file MCO2-7-e70661-s001.docx]

**TITLE: Varicella Zoster Virus Infection: Clinical Features, Molecular Pathogenesis, Treatment and Prevention**

**AUTHORS:** Lei Peng^#,a^, Honghao Song^#,a^, Tianying Li^#,g^, Yuqing Ma^#,f^, Chen Yan^d^, Yuhan Cao^g^, Kaiqiang Sun^d,e *^, Chaofeng Han^b,c *^, Hongbin Yuan^a *^

1. Department of Anesthesiology, Changzheng Hospital, Naval Medical University, Shanghai 200003, P.R. China;
2. Department of Histology and Embryology, Naval Medical University, Shanghai 200433, P.R. China；
3. National Key Laboratory of Immunity & Inflammation, Naval Medical University, Shanghai 200433, P.R. China;
4. Department of Orthopedic Surgery, Changzheng Hospital, Navy Medical University, Shanghai, 200003, P. R. China;
5. Department of Orthopedics, Naval Medical Center of PLA, Shanghai, 200052, P. R. China;
6. Nautical medicine experimental teaching demonstration center of educational institutions, Faculty of Naval Medicine, Navy Medical University, Shanghai, 200003, P. R. China;

g. School of Basic Medical Science, Naval Medical University, Shanghai 200433, P.R. China.

# These authors contributed equally to this work.

*** CORRESPONDING AUTHORS: Hongbin Yuan, Chaofeng Han, Kaiqiang Sun.**

| 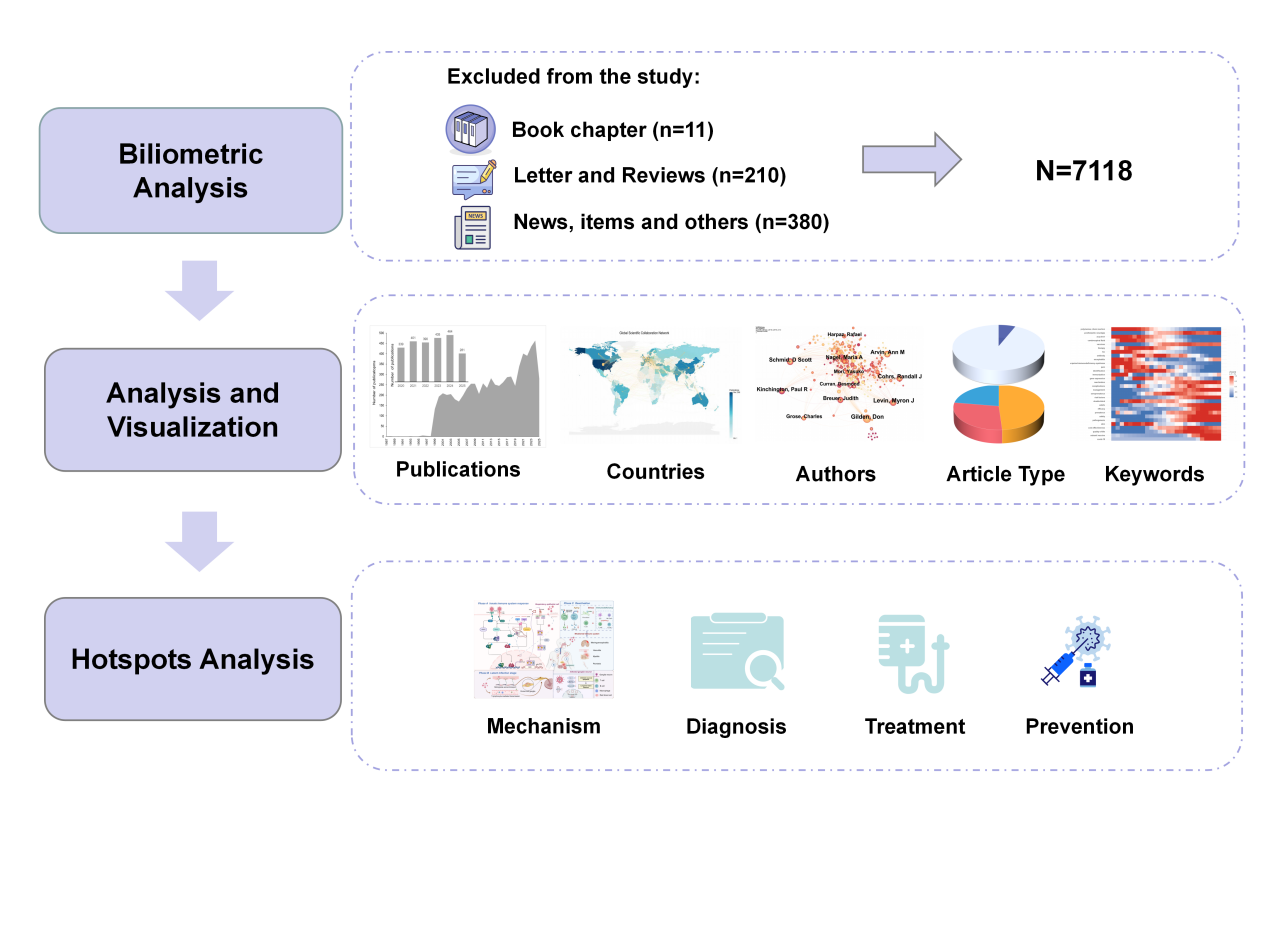 |
| --- |
| **Supplementary Figure 1**. **The process of literature screening, analysis and visualization, and hotspot analysis using bibliometrics.** |


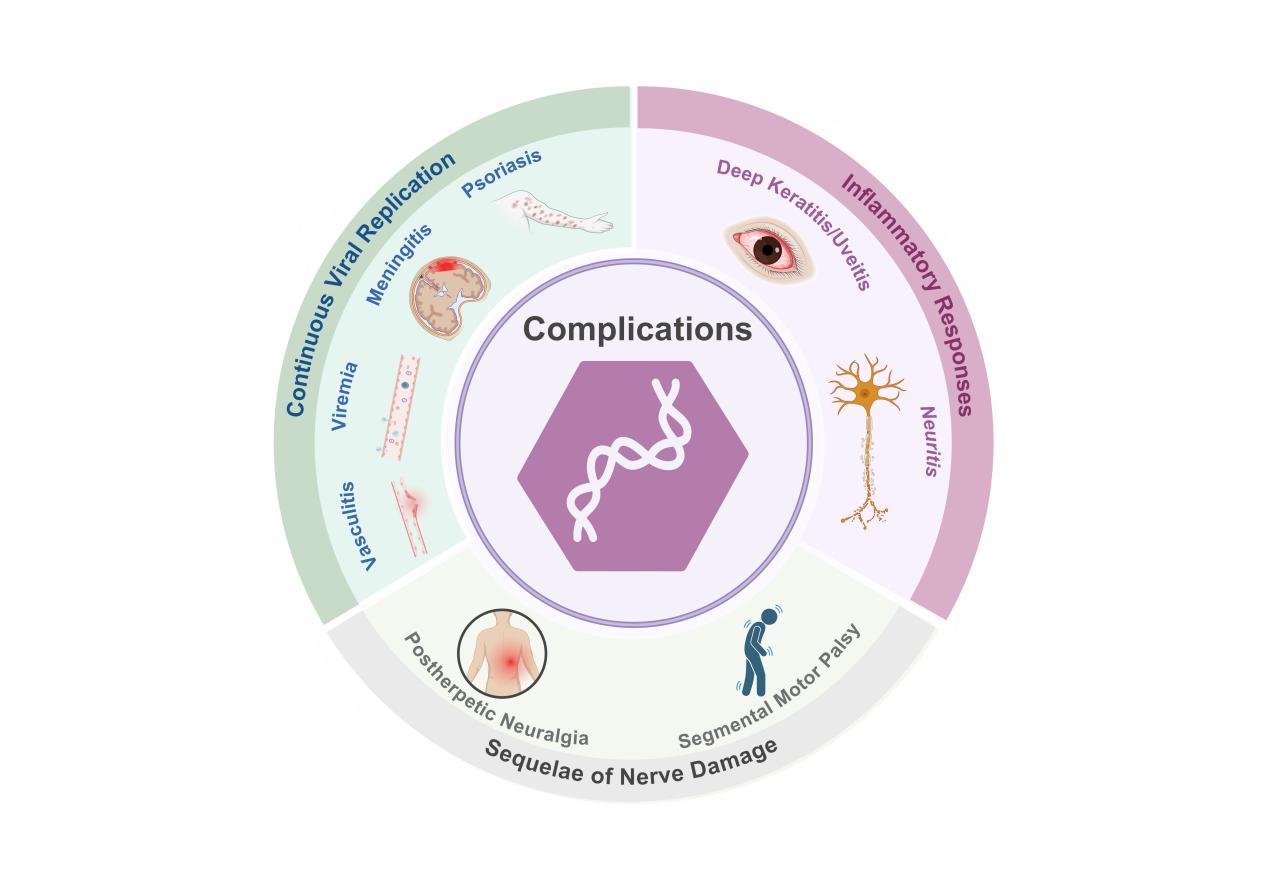


**Supplementary Figure 2**. **Schematic diagram of complications driven by different pathogenic mechanisms.**

| 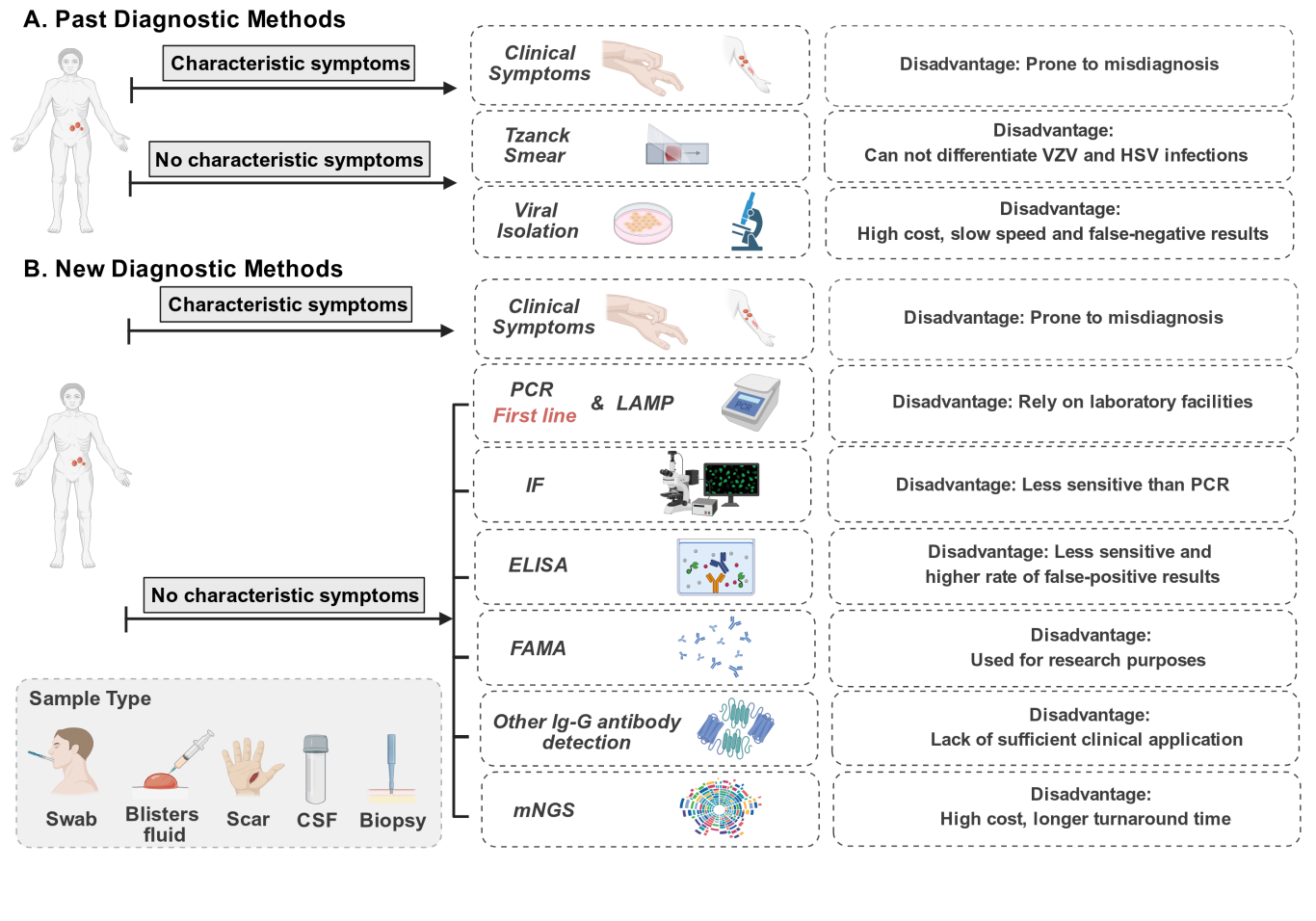 |
| --- |
| **Supplementary Figure 3**. **Schematic overview of diagnostic approaches for HZ.** (A) Historical diagnostic pathway: when patients present with characteristic symptoms such as pathognomonic dermatomal vesicular rash, diagnosis has traditionally relied on clinical assessment; in the absence of characteristic lesions, cytological (Tzanck smear) and virological (viral isolation) assays were used. (B) Contemporary diagnostic pathway: for cases with characteristic rash, clinical diagnosis remains important but is commonly complemented or confirmed by molecular testing. |
